# The ESCRT-0 Component HRS is Required for HIV-1 Vpu-Mediated BST-2/Tetherin Down-Regulation

**DOI:** 10.1371/journal.ppat.1001265

**Published:** 2011-02-03

**Authors:** Katy Janvier, Annegret Pelchen–Matthews, Jean-Baptiste Renaud, Marina Caillet, Mark Marsh, Clarisse Berlioz-Torrent

**Affiliations:** 1 Institut Cochin, Université Paris Descartes, CNRS (UMR 8104), Paris, France; 2 INSERM, U1016, Paris, France; 3 Cell Biology Unit, MRC Laboratory for Molecular Cell Biology, University College London, London, United Kingdom; Duke University Medical Center, United States of America

## Abstract

The Endosomal Sorting Complexes Required for Transport (ESCRT) machinery, a highly conserved set of four hetero-oligomeric protein complexes, is required for multivesicular body formation, sorting ubiquitinylated membrane proteins for lysosomal degradation, cytokinesis and the final stages of assembly of a number of enveloped viruses, including the human immunodeficiency viruses. Here, we show an additional role for the ESCRT machinery in HIV-1 release. BST-2/tetherin is a restriction factor that impedes HIV release by tethering mature virus particles to the plasma membrane. We found that HRS, a key component of the ESCRT-0 complex, promotes efficient release of HIV-1 and that siRNA-mediated HRS depletion induces a BST-2/tetherin phenotype. This activity is related to the ability of the HIV-1 Vpu protein to down-regulate BST-2/tetherin. We found that BST-2/tetherin undergoes constitutive ESCRT-dependent sorting for lysosomal degradation and that this degradation is enhanced by Vpu expression. We demonstrate that Vpu-mediated BST-2/tetherin down-modulation and degradation require HRS (ESCRT-0) function and that knock down of HRS increases cellular levels of BST-2/tetherin and restricts virus release. Furthermore, HRS co-precipitates with Vpu and BST-2. Our results provide further insight into the mechanism by which Vpu counteracts BST-2/tetherin and promotes HIV-1 dissemination, and they highlight an additional role for the ESCRT machinery in virus release.

## Author Summary

The release of HIV-1 particles requires a series of interactions between proteins encoded by the virus and key cellular components, including elements of the cellular membrane trafficking apparatus such as the Endosomal Sorting Complexes Required for Transport (ESCRT) machinery. This machinery is composed of four multiprotein complexes (ESCRT-0, -I, -II and –III) that are involved in the sorting of ubiquitinylated membrane proteins for lysosomal degradation. Gag, the major structural protein of HIV, recruits the ESCRT-I and III complexes to mediate the scission of budding virions. Following ESCRT-mediated scission of viral particles, the HIV-1 accessory protein Vpu promotes the release of the mature virions by counteracting a cellular restriction factor BST-2/tetherin that physically tethers viral particles to the plasma membrane of infected cells. Here we show that HRS, a component of the ESCRT-0 complex, is required for Vpu to efficiently modulate the expression of BST-2 and promote HIV-1 release, highlighting an additional role of the ESCRT machinery in virus production.

## Introduction

The assembly and release of HIV-1 particles requires a highly orchestrated series of interactions between proteins encoded by the virus and key cellular components, including elements of the cellular membrane trafficking apparatus and the ESCRT (Endosomal Sorting Complexes Required for Transport) machinery [1], [2], [3]. The ESCRT machinery was initially found to be involved in the recognition and sorting of membrane proteins to the internal vesicles of multivesicular bodies (MVBs)/late endosomes and the subsequent degradation of these cargoes in lysosomes. Subsequently, the ESCRT machinery was found to also play key roles in topologically related membrane scission reactions that occur during cytokinesis and the budding of a number of enveloped viruses, including HIV-1 [1], [4]. The ESCRT machinery comprises four multiprotein complexes ESCRT-0, -I, -II and -III whose sequential recruitment to endosomal membranes mediates (1) the formation (budding) and subsequent pinching off of intralumenal endosomal vesicles into MVBs and (2) the incorporation of ubiquitinylated membrane protein cargoes into the inwardly budding vesicles [4]. The ESCRT-0 protein HRS (also called hepatocyte growth factor-regulated tyrosine kinase substrate [HGS]) initiates this process by acting as a linker protein that binds, on the one hand, ubiquitinylated cargoes and, on the other, a PSAP motif in the ESCRT-I component TSG101 [4].

Topologically, the budding of HIV-1 particles at the plasma membrane resembles the budding of intralumenal vesicles into MVBs. Moreover, the HIV-1 Gag protein, the major structural protein of the virus, can recruit the ESCRT-I and ESCRT-III complexes through its C-terminal p6 domain (so called “late domain”) to mediate release of budding virions [5], [6], [7], [8]. A PTAP motif in the Gag p6 domain binds TSG101, mimicking the HRS-PSAP mediated recruitment of ESCRT-I. Failure to recruit ESCRT-I, and consequently ESCRT-III, leads to accumulation of viral budding intermediates at the surface of infected cells [5], [8]. In addition, a LYPX_n_L motif in the Gag p6 domain provides a second means to recruit the ESCRT machinery by binding the ESCRT-I and ESCRT-III interacting protein ALIX/AIP1 [7]. Although Gag mimics HRS in binding TSG101, HRS itself is not required for the HIV-1 budding and scission reactions [9]. Nevertheless, HRS was recently identified in a genome-wide siRNA screen for host cell factors involved in HIV-1 replication, suggesting an active role for ESCRT-0 [10].

In this study, we explored the role of HRS in the HIV-1 replication cycle. Using RNA interference and virological assays, we show that HRS is required for efficient release of HIV-1 particles. HRS function was not related to the described TSG101 activity in HIV-1 scission, but was related to the ability of the accessory viral protein Vpu to down-regulate the protein bone marrow stromal antigen 2 (BST-2 also called CD317/HM1.24/tetherin). BST-2 was recently identified as a cellular restriction factor that impedes the release of fully assembled HIV-1 by physically tethering particles to the plasma membrane of infected cells. Vpu counteracts this restriction and induces the down-regulation of BST-2 expressed at the cell surface. Vpu-induced cell surface down-regulation of BST-2 is associated with targeting of the cellular protein for degradation [11], [12], [13], [14], [15]. Here we show that BST-2 undergoes constitutive ESCRT-mediated lysosomal degradation. Moreover, HRS facilitates Vpu-induced BST-2 down-regulation and degradation, and therefore contributes to efficient HIV-1 release. Furthermore HRS co-precipitates with Vpu and BST-2. Altogether, our results highlight an additional role for the ESCRT machinery in HIV-1 release, and provide further understanding of the mechanism by which Vpu targets BST-2 for degradation.

## Results

### HRS is required for efficient HIV-1 release

**Figure 1:**
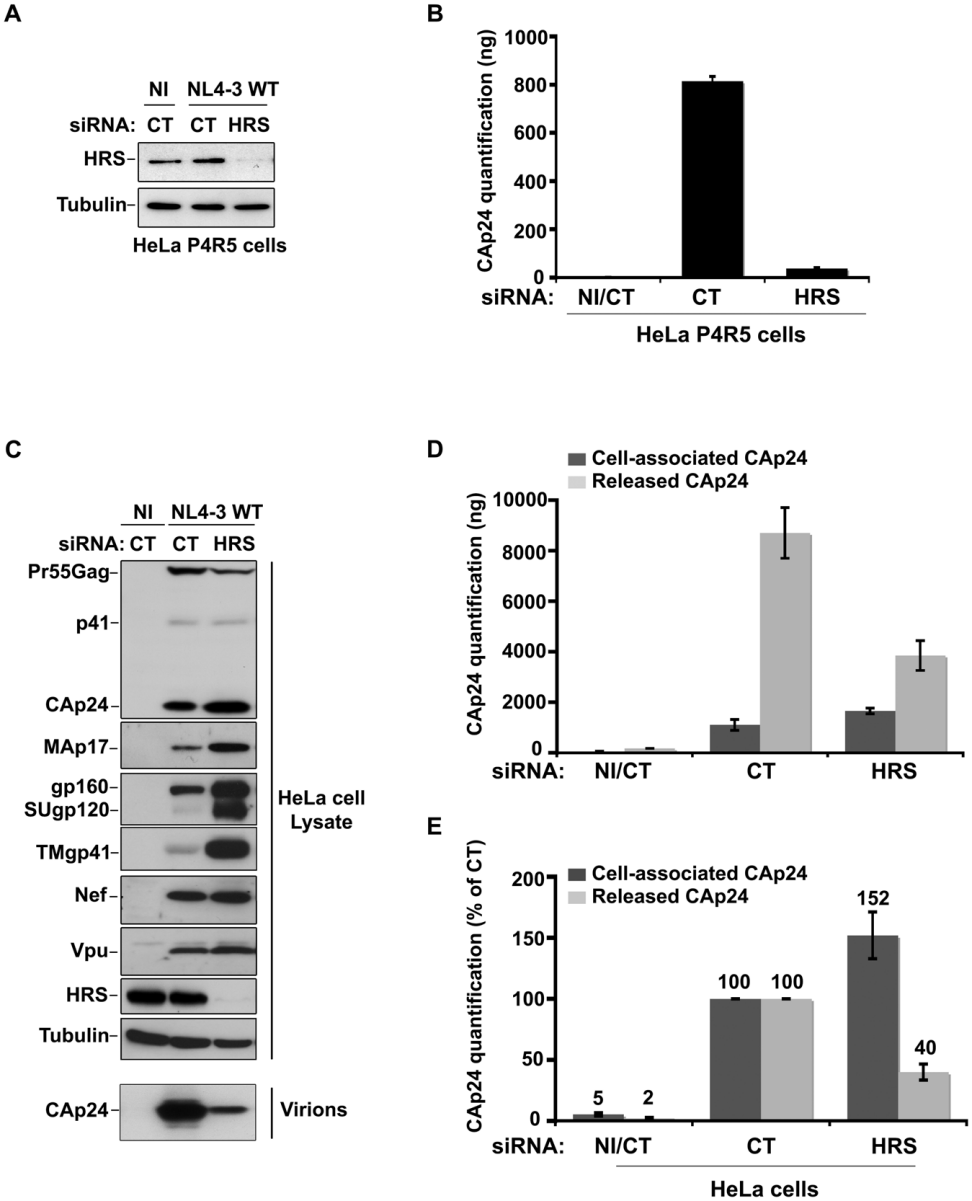
Depletion of HRS reduces the release of HIV-1 particles from infected cells. (**A–B**) **HRS depletion impairs HIV-1 propagation.** HeLa P4R5 MAGI cells transfected with either siRNA control (siRNA CT) or siRNA targeting HRS (siRNA HRS) were non-infected (NI) or infected with NL4-3 HIV-1 WT at a low MOI = 0.005 and left for 4 days to allow viral propagation. (**A**) Western-blot analysis of infected siRNA treated cells. Tubulin is the loading control. (**B**) HIV-1 propagation was scored by ELISA quantification of extracellular CAp24 present in the culture supernatants. (**C–E**) **HRS depletion reduces HIV-1 release.** HeLa cells transfected with either control siRNA (CT) or siRNA targeting HRS were infected with VSV-G-pseudotyped HIV-1 (NL4-3 WT). (**C**) Western-blot analysis of infected siRNA-treated cells (upper panels) and pelleted viruses (lower panel). Tubulin is the loading control. (**D**) CAp24 present within the cells (Cell-associated CAp24, dark-grey graph bars) and released from the infected cells (Released CAp24, light-grey graph bars) was measured by ELISA. The experiment was performed in duplicate. Bars represent the mean -/+ SD from each duplicate. (**E**) For HRS depleted cells, amounts of released and cell-associated CAp24 obtained by ELISA in (D) were normalized to those obtained for the corresponding control cells (set as 100%). Bars represent the mean −/+ SD from 4 independent experiments.

To investigate the role of the ESCRT-0 component HRS on HIV-1 replication, we specifically depleted HRS in HeLa cells and analysed the effect on HIV-1 propagation. We used a siRNA against HRS that has been described and validated in earlier studies ([16] Cf. Experimental procedures). By western-blot analysis, siRNA transfections into HeLa cells resulted in >95% reduction in HRS levels, as previously described ([16]; Figures 1A and C). We assessed the impact of HRS silencing on the ability of the NL4-3 strain of HIV-1 (NL4-3 WT) to propagate in the reporter cell line HeLa P4R5 (stably expressing the receptor CD4 and the coreceptor CCR5). The cells were either treated with a control siRNA (siRNA CT; referred to as control cells) or with siRNA targeting HRS (siRNA HRS), then infected with NL4-3 HIV-1 at a MOI of 0.005 and left for 4 days to allow the virus to propagate through the cultures. HIV-1 production was scored by ELISA quantification of the Gag product CAp24, reflecting the amount of virus present in the supernatant. Figure 1B shows that depletion of HRS caused a significant reduction in virus release, with <10% CAp24 detected in the supernatant of these cells compared to the control cells, suggesting that HRS is required for optimal HIV-1 replication.

As HIV-1 entry into HRS-depleted cells was not affected (Figure S1), we tested whether the reduction in HIV-1 propagation observed following HRS knock down was a consequence of decreased production of viral particles. To this end, HeLa cells were transfected with control siRNA (siRNA CT) or siRNA targeting HRS (siRNA HRS), and then infected with NL4-3 HIV-1 pseudotyped with VSV-G (NL4-3 WT) at an MOI = 0.5. VSV-G pseudotyping enables HIV-1 production to be monitored after a single round of infection. Forty-eight hours after infection the protein content of the cells was analyzed by western-blot (Figure 1C) and the virus released into the supernatant was assessed by western-blot and ELISA quantification of CAp24 (Figures 1D–E). Depletion of HRS (Figure 1C) did not significantly modify the maturation of Gag compared to the control cells. However, in HRS-depleted cells a clear accumulation of Env (gp160, SUgp120 and TMgp41) and of the processed Gag products, CAp24 and MAp17, was observed (Figures 1C–E). The expression levels of the viral accessory proteins Nef and Vpu were similar in both CT and HRS knock down cells. As these accessory proteins are not incorporated significantly into nascent viral particles, we postulated that the increased level of cell-associated Env and processed Gag products, CAp24 and MAp17, might be due to a decrease in HIV-1 release from HRS depleted cells. Quantification of the amount of CAp24 in the cell supernatants (Figures 1D and 1E) and western-blot analysis of viral preparations (Figure 1C, lower panel) confirmed that virus release was decreased by 60–70% in HRS knock down cells. These data indicate that HRS is required for efficient HIV-1 release from HeLa cells.

### HRS does not interfere with the Gag-related function of TSG101 in HIV-1 morphogenesis

**Figure 2:**
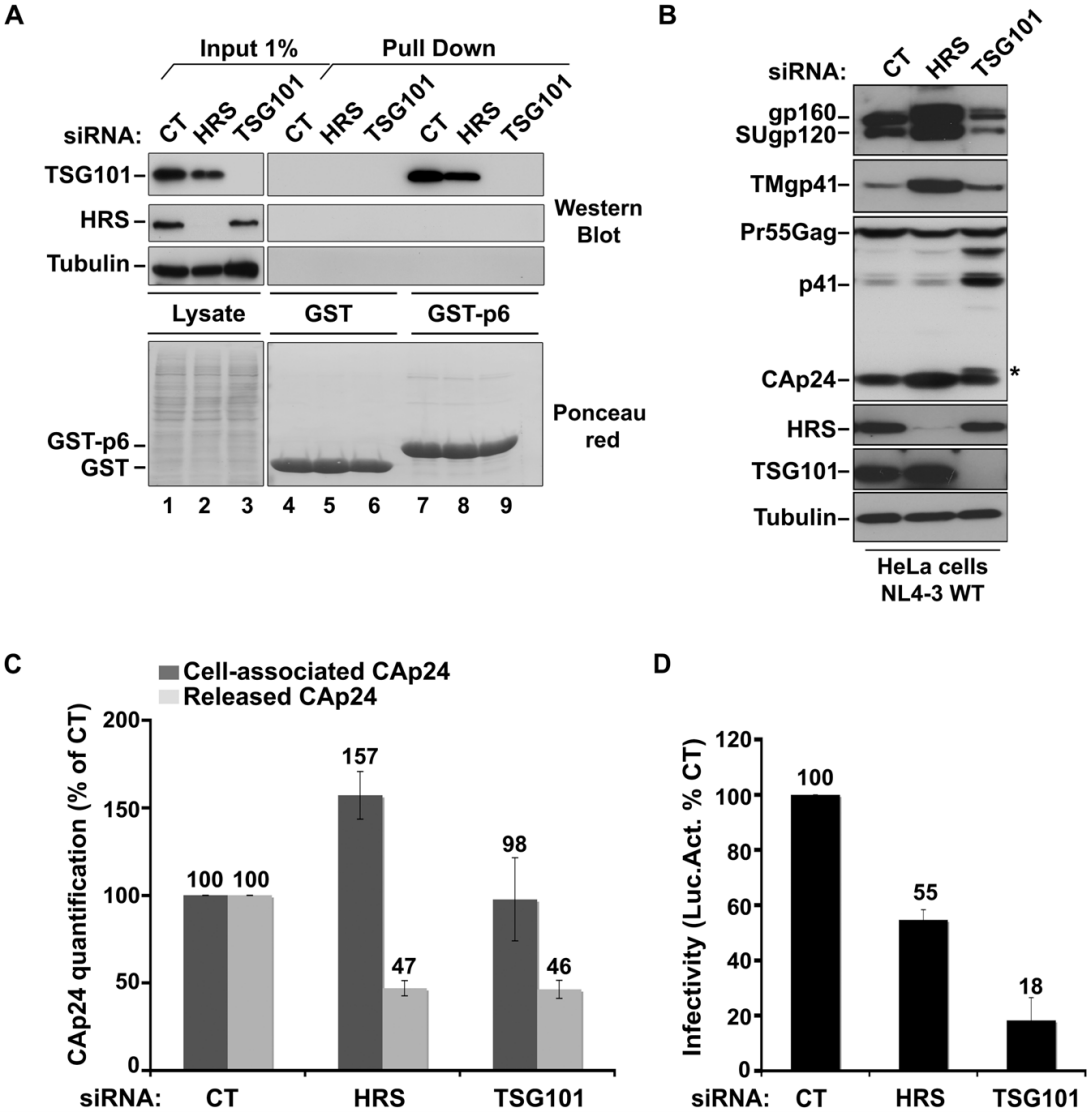
HRS depletion does not interfere with TSG101-mediated budding of HIV-1. (**A**) **Binding of TSG101 to the p6 domain of Gag.** Lysates of HeLa cells transfected with either control siRNA (CT) or siRNA targeting HRS or TSG101 were incubated with equal amounts of purified GST (lanes 4 to 6) or GST-p6 (lanes 7 to 9). TSG101 binding and HRS depletion were analysed by western blotting (upper panels). Tubulin is the loading control for cellular proteins. Lower panel: Ponceau red staining of the membrane used for western blotting. These data are representative of 2 independent experiments. (**B–D**) **Impact of HRS and TSG101 depletion on HIV-1 assembly.** HeLa cells transfected with either control siRNA (CT) or siRNA targeting HRS or TSG101 were infected with VSV-G-pseudotyped NL4-3 HIV-1 (NL4-3 WT). (**B**) Western-blot analysis of infected siRNA-treated cells. Tubulin is the loading control. Asterisk (*) shows the CA-SP1 Gag processing intermediate. (**C**) HIV-1 CAp24 present within the cells (Cell-associated CAp24, dark-grey graph bars) and released in the supernatant of the infected cells (Released CAp24, light-grey graph bars) was measured by ELISA. Values obtained for HRS or TSG101 depleted cells were normalized to those of the control cells set to 100%. Bars represent the mean −/+ SD from 3 independent experiments. (**D**) The virus titre was scored by infection of HeLa TZM-bl indicator cells, followed by luciferase activity quantification in the cells. The values obtained were normalized to the amount of released CAp24 quantified in (C). Bars represent the mean −/+ SD from 4 independent experiments.

To understand the mechanism by which HRS contributes to HIV-1 release, we assessed whether HRS depletion perturbed Gag recruitment of TSG101. To this end, we analysed the ability of a GST-p6 fusion construct, corresponding to the sub-domain of Gag bearing the PTAP motif involved in TSG101 interaction, to interact with TSG101 in control and HRS depleted cells. Figure 2A shows similar levels of binding of GST-p6 to TSG101 in control and HRS depleted cells (Figure 2A, compare lane 7 with lane 8). Similar results were obtained using a GST-Gag construct (Figure S2) suggesting that HRS is not required for p6-mediated binding of Gag to TSG101 and recruitment of ESCRT-I.

We then analyzed whether depletion of HRS and TSG101 led to similar alteration of virus production using the assay described above. As shown in Figure 2C, both HRS and TSG101 depletion reduced HIV-1 release from infected cells, as assessed by quantitative ELISA. However, western-blot analysis of cell-associated viral proteins suggested that HRS and TSG101 have different roles in HIV-1 production (Figure 2B). Depletion of TSG101, as well as expression of viruses mutated in the Gag PTAP motif, induced a profound alteration in Gag processing, characterized by an accumulation of the p41 and CA-SP1 Gag processing intermediates as previously described [5], [6]. No such alteration was observed upon depletion of HRS (Figures 1C and 2B). Infectious viral release from the siRNA-transfected cells was then scored using the indicator cell line HeLa TZM-bl, and the results were normalized to the amount of CAp24 present in the cell supernatants. As previously shown, particles produced by TSG101 depleted cells were mostly non-infectious due to defects in Gag processing (80% loss of infectivity compared to virions released from control cells) [5]. By contrast, the virions produced from HRS depleted cells exhibited <45% loss of infectivity compared to control viruses (Figure 2D). Interestingly, western-blot analysis of viral particles showed a decrease in envelope glycoprotein (Env) incorporation (Figure S3), which may explain this lower infectivity. HRS might also be required for the proper intracellular trafficking and processing of Env within infected cells. Therefore, HRS depletion might result in intracellular accumulation and incorporation of mature and unprocessed Env products unfit for HIV-1 infectivity (Figures 1C, 2B, 2D). Taken together, our data indicate that HRS is required for efficient HIV-1 release from HeLa cells, and its mode of action is distinct from that of TSG101 in HIV-1 budding.

### HRS depletion leads to accumulation of HIV-1 particles at the cell surface and in endosomes

**Figure 3:**
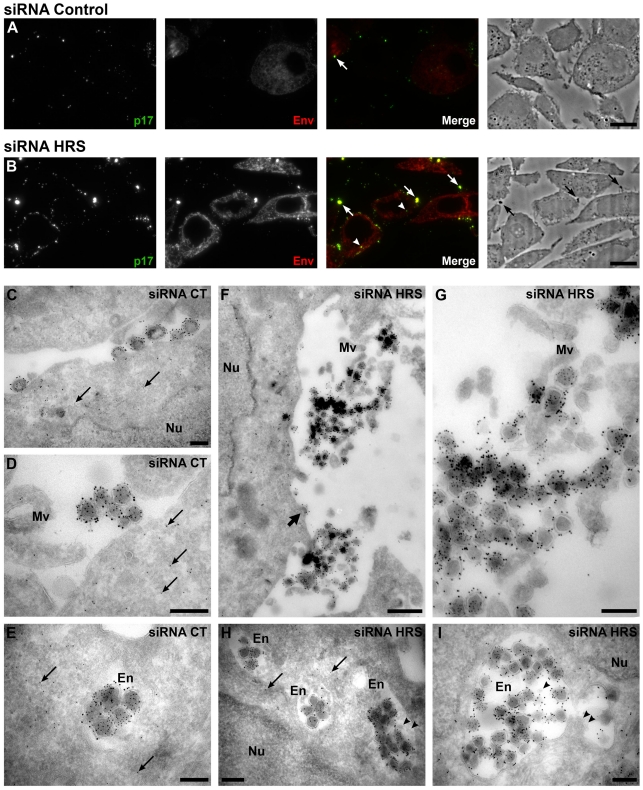
Silencing of HRS leads to accumulation of HIV-1 particles in at the cell surface and in endosomes. HeLa cells transfected with either siRNA control (A and C–E) or siRNA targeting HRS (B and F–I) were infected with VSV-G-pseudotyped HIV-1 for 48 hrs, fixed and prepared for cryosectioning. (**A**, **B**) **Immunofluorescence analysis of MAp17 and Env distribution on semi-thin cryosections.** Semi-thin (0.5 µm) cryosections were immunostained with mouse anti-MAp17 and anti-mouse-Alexa-488 (green) and 2G12 anti-HIV-Env and anti-human- Alexa-594 (red). MAp17 staining reveals fine spots probably representing virus particles at the surface of the cells, as well as larger clumps of virus staining (arrows) which can correspond to phase-dark structures at the surface of the cell profiles in the phase images shown in the right hand panels. Many more clumps of MAp17 staining were observed on the HRS-depleted cells (B). In addition, some virus staining was seen in intracellular vacuoles (arrowheads). Scale bars  = 10 µm. (**C–I**) **ImmunoEM analysis of Gag and Env distribution.** Ultrathin cryosections (50 nm) were labelled with anti-Gag p24/p55 (5 nm PAG) and anti-Env 2G12 (10 nm PAG). Infected cells show scattered 5 nm gold particles over the cytoplasm (thin arrows), but not over the nucleus (Nu). In the control cells immature and mature virus particles could be seen at the cell surface, individually or in small groups (C) or in small clumps (D) and, occasionally, in intracellular vacuoles (E). In cells treated with HRS siRNA, much larger clusters of HIV particles were seen at the cell surface or attached to microvillus-like cell surface protrusions (Mv) (F, the upper virus cluster is enlarged in G). In addition, intracellular vacuoles or endosomes (En) containing many virus particles were frequently seen (H, I). The thick arrow in F identifies a budding virus, and the arrowheads in H and I mark small intralumenal vesicles in the virus-containing endosomes. Scale bars in C–E and G–I  = 200 nm, F = 500 nm.

To further explore the mechanism by which HRS reduces HIV-1 release, siRNA treated HeLa cells infected with VSV-G pseudotyped NL4-3 HIV-1 were prepared for cryosectioning and electron microscopy (EM). Initially, immunofluorescence staining of semi-thin survey cryosections with antibodies against the viral proteins MAp17 and Env revealed a large number of HIV-infected cells (Figure 3A and 3B). The anti-MAp17 antibody 4C9, which detects only the cleaved Gag product MA, labelled small spots probably representing mature virions, as well as larger clumps of viruses, confirming that HRS depletion does not inhibit Gag processing and maturation of HIV-1 particles. Staining with antibodies against Env was observed over the endoplasmic reticulum, with juxta-nuclear patches of brighter staining, and some Env labelling co-localized with p17 stained viruses. On sections from HRS knockdown cells, the staining for p17 and Env was much stronger than on the control cells, with large clumps of viruses observed near the cell surface, sometimes attached via fine processes. Some virus was also observed in cytoplasmic vacuoles (Figure 3B).

To analyse the accumulations of virus particles in more detail, we stained ultrathin cryosections (50 nm) for p24/p55Gag and Env with protein A-gold (PAG) for EM. For both the control and HRS siRNA-treated samples, infected cells could be identified by scattered p55Gag labelling (5 nm PAG) over the cytoplasm, but not over nuclei, demonstrating specificity of the labelling (Figures 3C–E and 3H). Env was seen over the Golgi apparatus and associated small tubulo-vesicular membranes (Figure S4A and S4C). Virus particles were labelled with antibodies against both p24/p55Gag (5 nm PAG) and Env (10 nm PAG), showing that Env-containing virions were produced by control and HRS-depleted cells. On control cells, the virus particles were seen either singly or in small clusters at the cell surface (Figure 3C and 3D), and occasionally virus particles could also be observed in intracellular vacuoles (Figure 3E). On the HRS-depleted cells, much larger clusters of HIV particles were attached to the cell surface either directly or via microvillar protrusions (Figure 3F, the upper of these virus clusters is enlarged in Figure 3G). Some of these clusters contained >20 and sometimes as many as 80–100 virus particles. In addition, we observed intracellular vacuoles resembling endosomes containing many p24/p55Gag and Env-immunolabelled virus particles in some of the HRS-depleted cells (Figure 3H, 3I and S4F).

We confirmed these observations by counting the distribution of 669 and 395 virus particles in the HRS-depleted and control cells, respectively. On the HRS-depleted cells, 47% of the virus particles were in clusters of more than 20 particles, while in control cells about half of the viruses occurred alone or in groups of 2–4 particles, and no virus clusters contained more than 18 virions. In addition, for the HRS-depleted cells, 27% of the virus particles were seen in intracellular vesicles, while for the control cells only 7% of virus particles were in endosomes. The large extracellular virus clusters and accumulation of virus particles in endosomes in the HRS-depleted cells was reminiscent of the phenotype observed in BST-2/tetherin-expressing cells infected with Vpu-defective HIV-1 strains [13], [15]. This suggests that depletion of HRS in HeLa cells leads to tethering of viral particles at the cell surface and their subsequent endocytosis, and that HRS knock down may affect the trafficking of BST-2.

### The ESCRT machinery targets BST-2 for degradation

**Figure 4:**
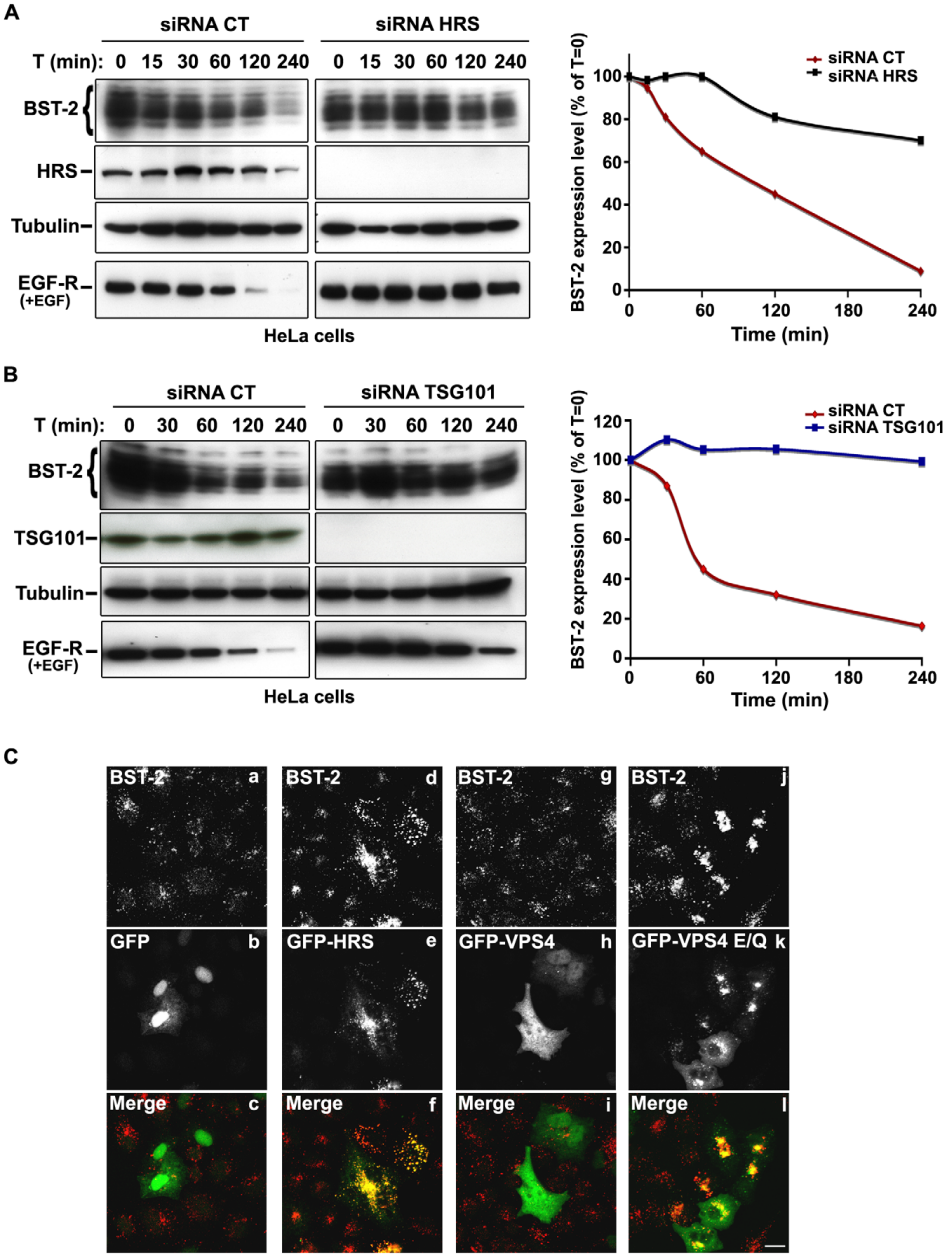
The ESCRT machinery sorts BST-2 for degradation. (**A**) **Analysis of BST-2 turnover following HRS depletion.** HeLa cells were transfected with either control siRNA (CT) or siRNA targeting HRS. Four days later the cells were incubated with cycloheximide (10 µg/ml) and, where indicated, with EGF for the times indicated above each lane. Cells were lysed and equivalent amounts of each sample (40 µg of protein) were analysed using western blotting with antibodies against BST-2, HRS, EGF-Receptor and tubulin as a loading control. For each sample, BST-2 signal intensity was quantified using the “Multi Gauge” software and used to calculate the relative amount of BST-2 at each time point. Values at time 0 were set to 100% in the graph shown on the right. The data presented are representative of 3 independent experiments. (**B**) **Analysis of BST-2 turnover upon TSG101 depletion**. HeLa cells transfected with either control siRNA (CT) or siRNA targeting TSG101 were treated 3 days after transfection as described above (A). The data presented are representative of 2 independent experiments (see also Figure S5). (**C**) **Effect of overexpression of HRS and dominant negative VPS4 mutant on BST-2 distribution**. HeLa cells transfected with plasmids encoding GFP, GFP-HRS, GFP-VPS4, or GFP-VPS4-E/Q for 16 hrs and permeabilized before fixation and staining with mouse polyclonal anti-BST-2. Colocalisation was assessed by confocal microscopy. Bar: 10 µm.

The HIV-1 accessory protein Vpu counteracts the cellular restriction factor BST-2 to promote HIV-1 release. BST-2 physically tethers newly formed viral particles at the surface of infected cells, preventing their release. Vpu relieves this restriction by down regulating cell surface expression of BST-2. In addition, Vpu promotes the degradation of BST-2 [11], [12], [13], [14], [15], [17]. Based on the EM observations above, we postulated that HRS might contribute to the process of Vpu inhibition of BST-2 restriction. As the ESCRT machinery sorts ubiquitinylated membrane proteins for lysosomal degradation, we analysed whether HRS, and by extension the MVB pathway, participates in the degradation of BST-2. HeLa cells were transfected with siRNAs targeting HRS or TSG101 and the turnover of BST-2 was monitored after incubating the cells in growth medium containing cycloheximide. We observed that almost 90% of BST-2 was degraded in 4 hours in cells transfected with control siRNA (Figure 4A; siRNA CT). By contrast, in HRS depleted cells, the half-life of BST-2 was prolonged, with only 30% of the initial BST-2 pool degraded after 4 hours (Figure 4A; siRNA HRS). Western-blot and immunofluorescence analysis revealed increased BST-2 expression in HRS depleted cells compared to the control cells, consistent with stabilization of the protein (Figure S5A and C). We also investigated the role of TSG101 in the degradation of BST-2. As TSG101 depletion is achieved more rapidly than HRS depletion (∼2 days and 4 days, respectively), the cells were harvested 72 hours after siRNA transfection (Figure 4B). As observed with HRS knock down, loss of TSG101 also stabilized BST-2 expression (less than 10% degradation in 4 hrs; Figure 4B). This stabilization was also associated with a striking accumulation of BST-2 within intracellular structures that partially co-labelled for HRS (Figure S5B and C). As an internal control, we monitored the turnover of EGF receptors (EGF-R) following EGF stimulation. Upon ligand binding, EGF-R is targeted to MVBs prior to lysosomal degradation [18], [19]. Consistent with these previous studies, knock down of HRS (Figure 4A) or TSG101 (Figure 4B) inhibited EGF-induced EGF-R degradation compared to control cells (Figures 4A–B, lower panels). These data show that HRS and TSG101 are involved in the sorting of BST-2 for degradation in the MVB/lysosomal pathway.

To obtain further evidence for the involvement of the ESCRT machinery in BST-2 trafficking, we analysed the effect of over-expressing HRS, or expressing a dominant negative mutant of the v-ATPase VPS4 (VPS4-E_223_/Q), on BST-2 expression. Over-expression of HRS was shown to negatively perturb the ESCRT pathway [18]. VPS4 is an AAA-ATPase involved in recycling the ESCRT machinery by facilitating its dissociation from endosomal membranes [20]. Mutation of E223 to Q blocks ATP hydrolysis and renders VPS4-E_223_/Q a dominant-negative inhibitor of the ESCRT pathway [20]. Immunofluorescence microscopy showed that in cells expressing GFP-HRS, BST-2 accumulated within enlarged endosomal structures that co-labelled with GFP-HRS, compared to the neighbouring cells in which BST-2 is expressed in discrete punctuate structures (Figure 4C, panels d-f). Similarly, expression of the VPS4 dominant negative mutant resulted in a striking accumulation of BST-2 (Figure 4C, panels j-l), suggesting that expression of VPS4-E_223_/Q as well as over-expression of HRS inhibited BST-2 degradation compared to control cells expressing wild type VPS4 or GFP alone (Figure 4C). Together, our results indicate that BST-2 is rapidly turned over in HeLa cells and that the ESCRT machinery is required for its efficient degradation.

### Impact of HRS depletion on the release of Vpu-defective HIV-1

**Figure 5:**
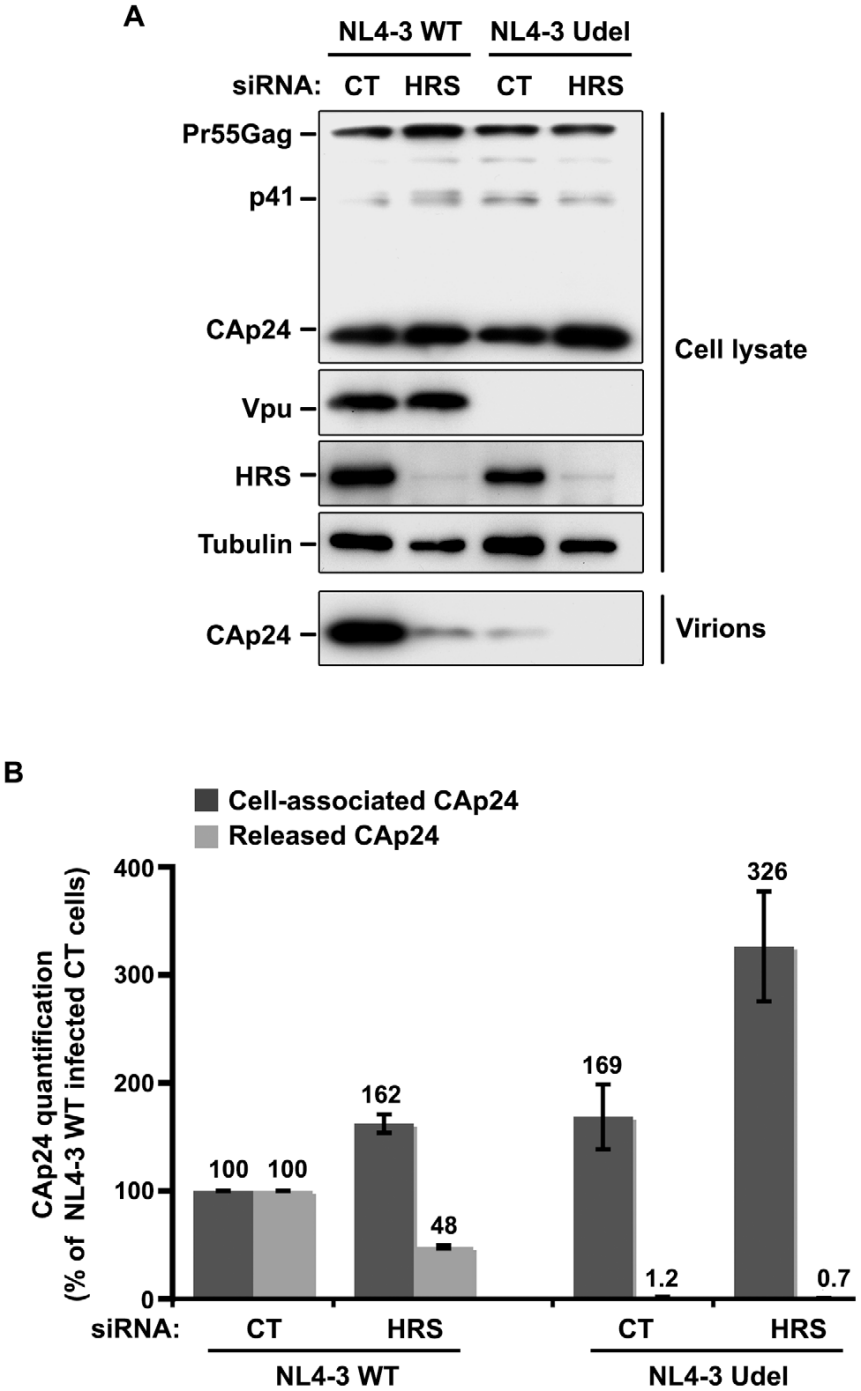
Impact of HRS depletion on the release of Vpu defective HIV-1 particles. HeLa cells were transfected with either control siRNA (CT) or siRNA targeting HRS and infected with either VSV-G pseudotyped wt NL4-3 HIV-1 (NL4-3 WT) viruses or VSV-G pseudotyped Vpu-defective NL4-3 (NL4-3 Udel) viruses. (**A**) Western-blot analysis of infected siRNA-treated cells (upper panels) and pelleted virus (lower panel). (**B**) CAp24 present within the cells (Cell-associated CAp24, dark-grey graph bars) and released in the supernatant of the infected cells (Released CAp24, light-grey graph bars) was measured by ELISA. The values were normalized to those obtained for control cells (CT) infected with WT viruses set to 100%. Quantifications were performed in duplicate. Bars represent the mean −/+ SD from 3 independent experiments.

As Vpu expression promotes the release of virus particles from restrictive BST-2-positive cells [13], [15], we analysed the effect of HRS depletion on the production of Vpu-defective HIV-1. To this end, HeLa cells were transfected with control siRNA or siRNA targeting HRS and then infected with either VSV-G pseudotyped wt NL4-3 HIV-1 (NL4-3 WT) or VSV-G pseudotyped Vpu-defective NL4-3 HIV-1 (NL4-3 Udel) at an equivalent MOI (MOI = 0.5). The cell proteins were then analysed by western-blot and the viral particles released into the cell supernatants measured by CAp24 ELISA and analysed by western-blot. As previously described [13], [15], the release of Vpu-defective HIV-1 particles from restrictive cells was poor compared to wild type virus (Figure 5). Interestingly, in HRS knock down cells, the release of Vpu-deleted HIV-1 was also lower (∼40% less virus) compared to the control cells (Figure 5A, lower panel and Figure 5B). As observed for WT viruses, the reduced release of Vpu-defective HIV-1 observed in HRS depleted cells was associated with increased amounts of cell-associated Gag/CAp24 (Figure 5B). These results suggest cooperation between Vpu and HRS to promote efficient HIV-1 release.

### Impact of HRS depletion on HIV-1 release from cells lacking BST-2

**Figure 6:**
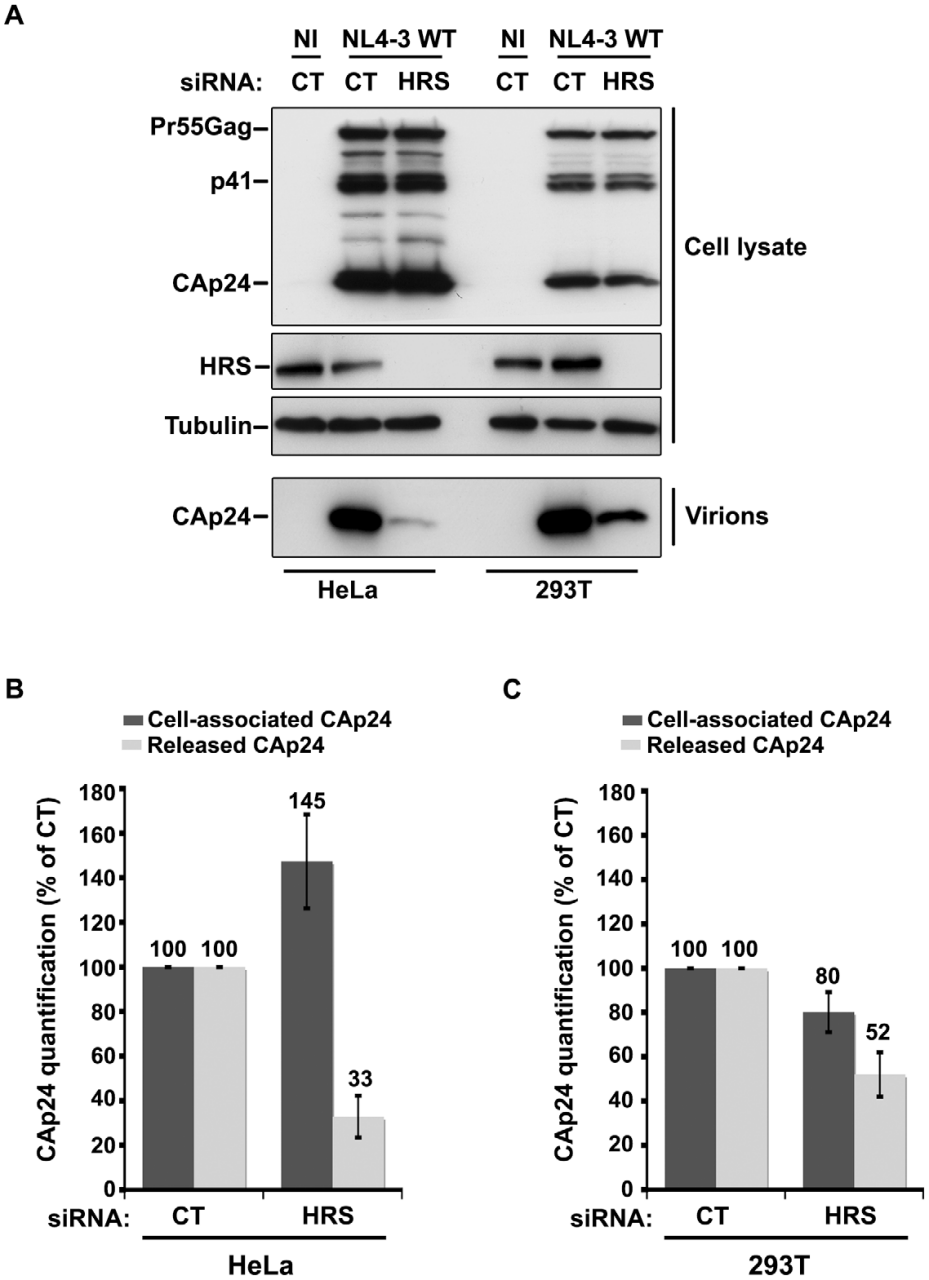
Effect of HRS depletion on the release of HIV-1 particles from non-restrictive HEK 293T cells. HeLa cells and HEK 293T cells transfected with either control siRNA (CT) or siRNA targeting HRS were infected with VSV-G-pseudotyped NL4-3 HIV-1 (NL4-3 WT). (**A**) Western-blot analysis of infected siRNA-treated cells (Upper panels) and viral particles released in the supernatant (Lower panel). Tubulin is the loading control. (**B**, **C**) CAp24 present within the cells (Cell-associated CAp24, dark grey graph bars) and released from the infected cells (Released CAp24, light-grey graph bars) was measured by ELISA quantification. The experiment was performed in duplicate. For each cell type, values obtained for HRS depleted cells were normalized to those obtained for the control cells (set as 100%). Bars represent the mean −/+ SD from 3 independent experiments.

Cells such as PBMC, HEK 293T, COS, HT1080, and HOS do not normally express BST-2. These cells are referred to as ‘non-restrictive’ and release the Vpu-defective HIV-1 as efficiently as wild type viruses [13], [15], [21]. To further explore the role of HRS in the BST-2 restriction of HIV-1 release, we compared the effect of HRS depletion on HIV-1 production in non-restrictive HEK 293T cells and restrictive HeLa cells (Figure 6). RNAi mediated HRS knock down was similar in HEK 293T and HeLa cells (>95%; Figure 6). In HeLa cells (Figure 6A and 6B), HRS depletion inhibited the release of HIV-1 and induced an accumulation of cell-associated CAp24 as shown before. In the HEK 293T cells, a reduced amount of CAp24 was detected in the supernatant, but this was paralleled by a decrease in the amount of cell-associated CAp24 (Figure 6A and 6C) compared to the control. Thus, in HEK 293T cells the decreased p24 release most likely reflects reduced HIV-1 infection rather than a defect in virus release (Figure 6C).

**Figure 7:**
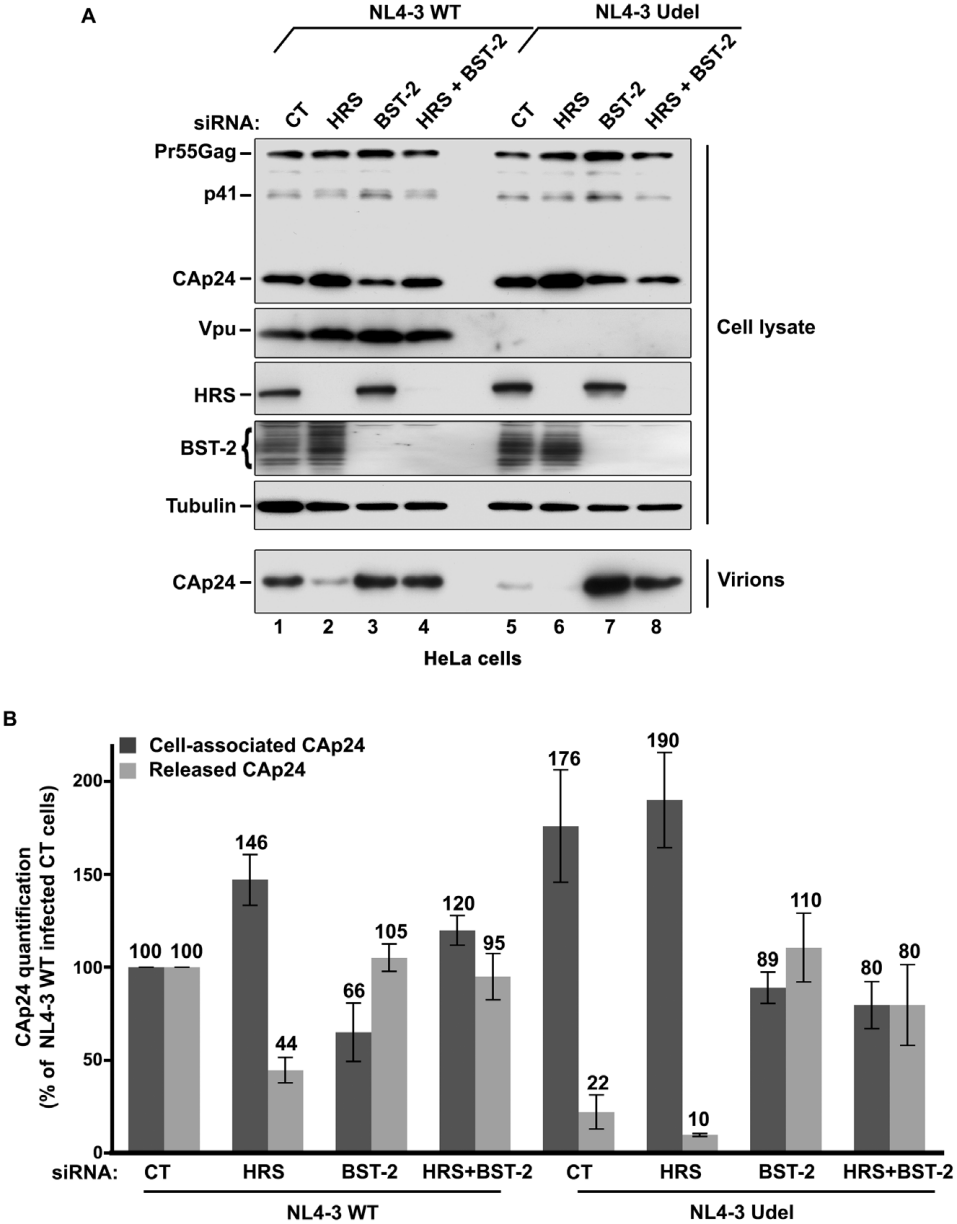
HRS depletion does not alter the release of HIV-1 particles in HeLa cells depleted for BST-2. HeLa cells transfected with either control siRNA (CT) (lanes 1 and 5) or siRNA targeting HRS (lanes 2 and 6), BST-2 (lanes 3 and 7) or both HRS and BST-2 (lanes 4 and 8). The cells were then infected with either VSV-G pseudotyped wt NL4-3 HIV-1 (NL4-3 WT) or VSV-G pseudotyped Vpu-defective NL4-3 (NL4-3 Udel) viruses at a MOI of 0.5. (**A**) Western-blot analysis of infected siRNA-treated cells (Upper panels) and viral particles released in the supernatant (Lower panel). Tubulin is the loading control. (**B**) CAp24 present within the cells (Cell-associated CAp24, dark grey graph bars) and released from the infected cells (Released CAp24, light-grey graph bars) was measured by ELISA. The values were normalized to those obtained for control cells (CT) infected with WT viruses set to 100%. Bars represent the mean -/+ SD from 3 independent experiments.

To circumvent the caveats caused by the lower sensitivity of HRS depleted HEK 293T cells to HIV-1 infection, we analysed the outcome of HRS depletion in HeLa cells depleted for BST-2 (Figure 7). HeLa cells were transfected with CT siRNA or siRNA targeting HRS and BST-2 alone or together and then infected with either VSV-G pseudotyped wt NL4-3 HIV-1 (NL4-3 WT) or VSV-G pseudotyped Vpu-defective NL4-3 HIV-1 (NL4-3 Udel) at an equivalent MOI (MOI = 0.5). As previously described, the release of Vpu-defective HIV-1 was rescued in cells depleted for BST-2 compared to the control cells (Figure 7A lower panel; compare lane 7 to lane 5, see Figure 7B), consistent with a restriction of Vpu-defective virus release by BST-2 [13], [15]. Interestingly HRS depletion did not affect the release of NL4-3 wt viral particles in BST-2 depleted cells (lower panel, compare lane 4 to lane 2; Figure 7B), as similar amounts of virus were produced from these cells compared to the control cells (compare lane 4 to lane 1). Similarly, release of NL4-3 Udel viruses was no longer affected by HRS depletion in HeLa cells devoid of BST-2 (Figure 7, lower panel, compare lane 8 to lane 6, see Figure 7B). These results support the notion that HRS does not play a major role in HIV-1 release from non-restrictive cells and suggest that HRS may contribute to the activity of Vpu in counteracting BST-2/tetherin mediated restriction of virus release.

### HRS is required for the Vpu-induced down regulation of BST-2

**Figure 8:**
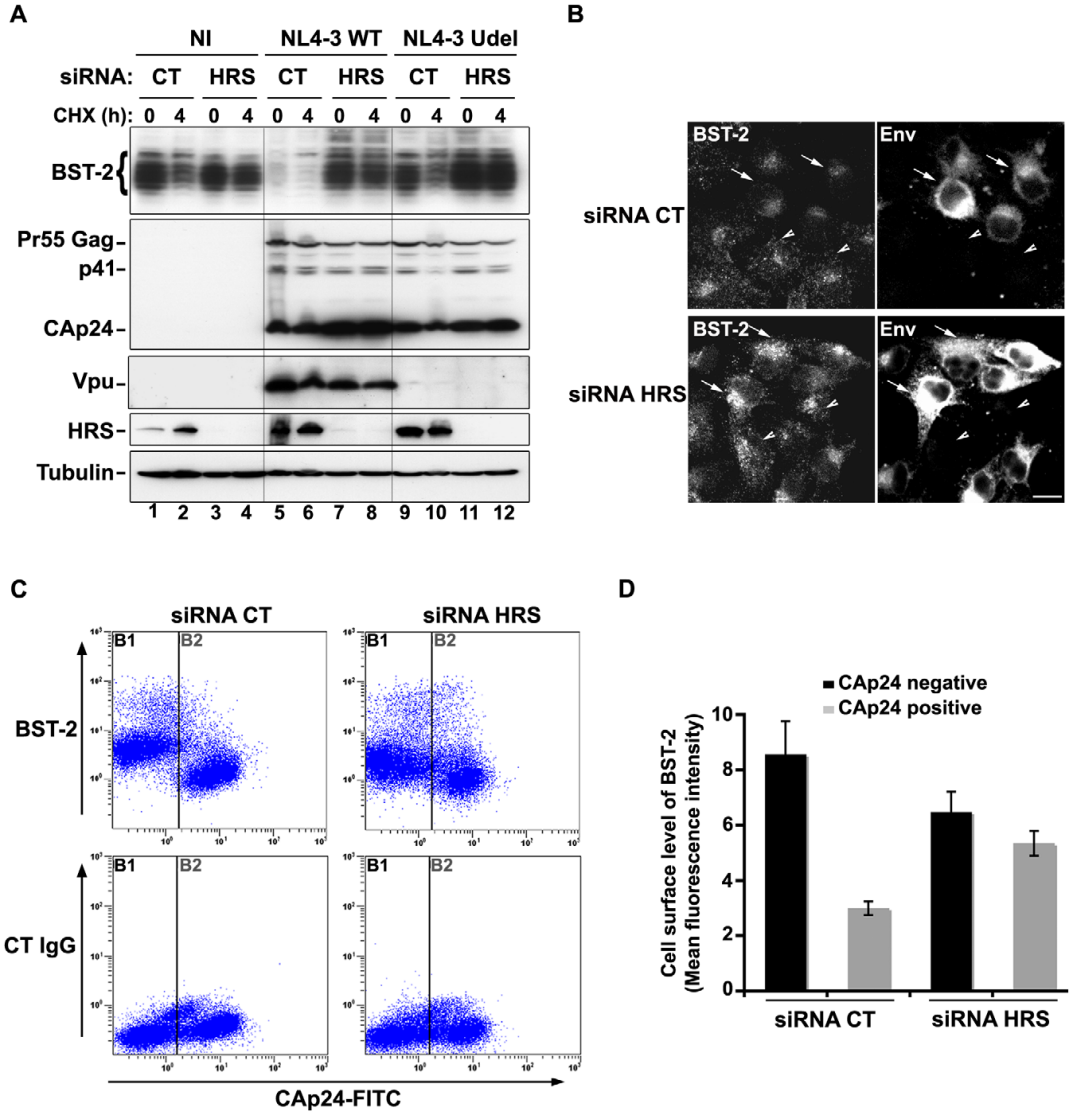
HRS is required for Vpu-induced BST-2 degradation and cell surface down-regulation. (**A–B**) **HRS depletion prevents Vpu from targeting BST-2 for degradation.** (**A**) HeLa cells transfected with either control siRNA (CT) or siRNA targeting HRS were infected with either VSV-G pseudotyped wt NL4-3 HIV-1 (NL4-3 WT) or VSV-G pseudotyped Vpu-defective NL4-3 (NL4-3 Udel) viruses at a MOI of 1, or left non-infected (NI). Forty-eight hours after infection, some of the cells were incubated with cycloheximide for 4 h and lysed. Cell lysates were analysed by western-blot. Tubulin is the loading control. These data are representative of 2 independent experiments. (**B**) HeLa cells transfected with either control siRNA (CT) or siRNA targeting HRS were infected with VSV-G pseudotyped NL4-3 HIV-1 (NL4-3 WT) and processed for immunolabelling with mouse polyclonal BST-2 and human HIV-1 Env (2G12) antibodies. Cells were imaged by confocal microscopy. Env staining discriminates between infected cells (arrows) and non-infected cells (arrowheads). Bar: 10 µm. (**C–D**) **HRS depletion impairs Vpu-induced cell surface down regulation of BST-2.** HeLa cells transfected with either control siRNA (CT) or siRNA targeting HRS were infected with VSV-G pseudotyped NL4-3 HIV-1 at a MOI of 0.5. Forty-eight hours later the cells were stained with rabbit polyclonal antibody against BST-2 (BST-2; upper panels) or rabbit polyclonal IgGs as an isotype control (CT IgG; lower panels), followed by a staining with a Cy5-conjugated donkey anti-rabbit antibody. The cells were then fixed, permeabilized and stained for Gag using a FITC-conjugated mouse monoclonal anti-CAp24. The cells were then processed for flow cytometry analysis. (**C**) Dot plot. Vertical lines indicate the gates set using non-infected cells stained as indicated. Gate B1 represents non infected cells and gate B2: infected-cells. (**D**) Bar graph representation of cell surface level of BST-2 in CAp24 negative cells (black bars) and CAp24 positive cells (grey bars) for each siRNA condition. Values are expressed as the Mean Fluorescence Intensity (MFI) on gate B1 (black bars) and B2 (grey bars) respectively. Error bars represent the –/+ SD from 3 independent experiments.

We observed that HRS is involved in the constitutive degradation of BST-2 (Figure 4A) and appears to promote HIV-1 release from restrictive cells (Figures 1C–E). We therefore tested whether HRS is required for Vpu induced BST-2 degradation. RNAi-treated HeLa cells were infected with either VSV-G pseudotyped wt NL4-3 (NL4-3 WT) or Vpu-deleted NL4-3 (NL4-3 Udel) viruses at a MOI of 1 to infect the majority of the cells. Forty-eight hours after infection, the cells were incubated in medium containing cycloheximide to monitor the degradation of BST-2 with time (Figure 8A). As described above (Figure 4), the level of BST-2 decreased in control cells in 4 hours of treatment with cycloheximide. By contrast, BST-2 levels were stabilized in HRS depleted cells over 4 hours (Figure 8A, compare lanes 1–2 with lanes 3–4). A similar pattern was observed in cells infected with Vpu-defective viruses (lanes 9–10 to 11–12). In control cells infected with NL4-3 WT, BST-2 was barely detectable at time 0, consistent with Vpu-enhanced degradation of BST-2 (lanes 5–6). Interestingly, in HRS depleted cells, BST-2 levels were similar to those in uninfected cells or cells infected with Vpu-defective viruses at time 0 and remained stable during 4 h of treatment with cycloheximide (Figure 8A, lanes 7–8). Immunofluorescence staining showed that, in control cells, HIV-1 infection (revealed by staining for Env) led to reduced BST-2 staining, compared to neighbouring non-infected cells, whereas BST-2 labelling was comparable in infected and non-infected cells when HRS was depleted (Figure 8B). These data show that HRS depletion impedes the ability of Vpu to target BST-2 for degradation. Taken together, our data indicate that Vpu-induced BST-2 degradation involves the ESCRT/MVB pathway.

Although Vpu expression can induce BST-2 degradation, the mechanism by which Vpu inhibits BST-2 restriction of HIV-1 release is unclear. Treatment of cells with the v-ATPase inhibitor bafilomycin A1, which inhibits both endosomal sorting and lysosomal function, can restore BST-2 cell surface levels in cells expressing Vpu, most likely due to the recycling of BST-2 [17]. We thus wondered whether depleting HRS counteracts Vpu-mediated down regulation of cell surface BST-2. To answer this question, siRNA transfected HeLa cells were infected with VSV-G pseudotyped wt NL4-3 HIV-1 (NL4-3 WT) at a MOI of 0.5 so that approximately 50% of the cells were infected. Cell surface levels of BST-2 were assessed by flow cytometry (Figures 8C–D and Table S1). Intracellular staining of CAp24 (CAp24-FITC) was used to distinguish non-infected cells (Figure 8C–D: gate B1 and black bars) from infected cells (Figure 8C–D: gate B2 and grey bars). In control cells (siRNA CT) infected with WT NL4-3 HIV-1, BST-2 cell surface expression decreased by ≥60% compared with non-infected cells, consistent with previous studies [15], [22]. Interestingly, the Vpu-induced down regulation of cell surface BST-2 was impaired upon depletion of HRS since no significant decrease of cell surface BST-2 was observed on infected HRS depleted cells (less than 20% decrease) compared to non-infected HRS depleted cells (Figure 8C–D and Table S1). Our data suggest that disruption of BST-2 sorting in endosomes by HRS depletion diminishes the ability of Vpu to down-regulate cell surface BST-2, most likely by allowing internalized BST-2 to recycle to the cell surface.

### The v-ATPase VPS4 is required for Vpu-induced cell surface down regulation of BST-2

**Figure 9:**
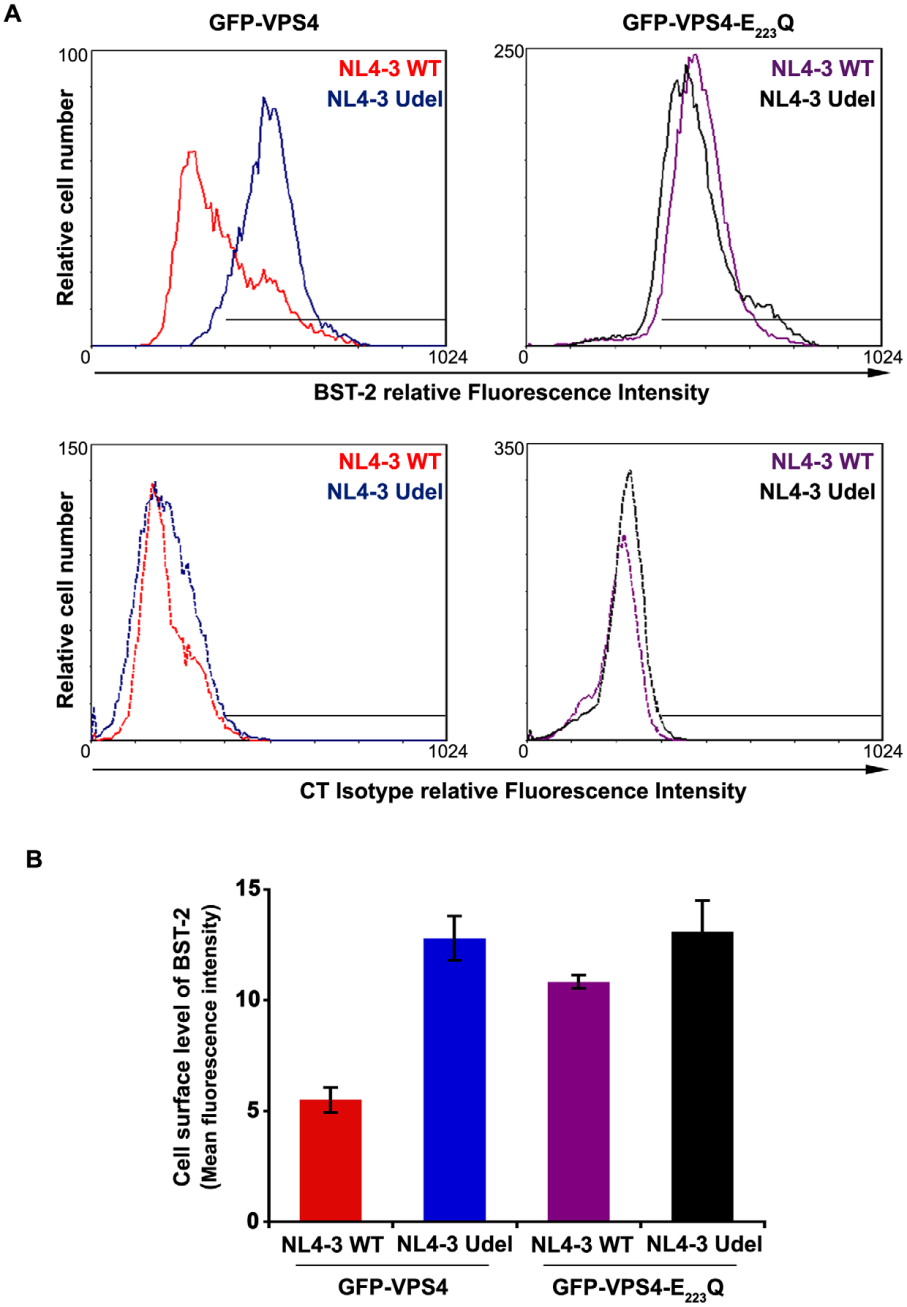
Overexpression of a dominant negative VPS4 mutant impairs Vpu-induced BST-2 cell surface down-regulation. (**A**) HeLa cells were transfected with plasmids encoding GFP-VPS4 or GFP-VPS4-E_223_/Q along with plasmids encoding the provirus NL4-3 wt (red and purple histograms) or Vpu-defective NL4-3 (NL4-3 Udel; blue and black histograms). Forty-eight hours later, the cells were co-stained with a mouse monoclonal antibody against BST-2 (BST-2; upper panels) or isotype control mouse IgG1 (CT Isotype; lower panels) and a human monoclonal antibody against Env. Cells were then co-stained with Alexa 647-conjugated donkey anti-mouse and PE-conjugated donkey anti-human antibodies, fixed, and processed for flow cytometry analysis. Histograms represent the relative cell number vs. BST-2 or Isotype IgG fluorescence intensity for the GFP HIV-1 positive cells (GFP + PE positive cells). Dotted histograms are the isotype controls. This experiment is representative of 3 independent experiments. (**B**) Bar graph representation of cell surface BST-2 in cell expressing (i) GFP-VPS4 wt along with HIV-1 NL4-3 WT (red bar) or HIV-1 NL4-3 Udel (blue bar) or (ii) GFP-VPS4-E_223_/Q CAp24 along with HIV-1 NL4-3 WT (purple bar) or HIV-1 NL4-3 Udel (black bar). Values are expressed as the Mean Fluorescence Intensity (MFI) obtained for BST-2 staining minus MFI values obtained for the corresponding isotype control. Error bars represent the –/+ SD from 3 independent experiments.

To obtain further evidence that the mechanism by which Vpu counteracts BST-2 involves the ESCRT machinery, we analysed the effect of over-expressing a dominant negative mutant of the AAA-ATPase VPS4 (VPS4-E_223_/Q) on Vpu-induced down-regulation of cell surface BST-2. HeLa cells were co-transfected with plasmids encoding wt VPS4 or VPS4-E_223_/Q fused to GFP along with the plasmid encoding the provirus wt NL4-3 HIV-1 (NL4-3 wt) or Vpu-defective NL4-3 HIV-1 (NL4-3 Udel) as a control. Cell surface levels of BST-2 were assessed by flow cytometry on GFP positive infected cells (surface staining of Env was used to identify HIV-1 expressing cells) (Figure 9). In cells expressing wt VPS4, a marked decrease of cell surface BST-2 was observed in cells expressing HIV-1 NL4-3 wt compared to cells expressing Vpu-defective viruses (Figure 9A, upper left panel and Figure 9B), consistent with Vpu-induced down–regulation of cell surface BST-2 [11], [12], [15], [17], [22]. Interestingly, expression of VPS4-E_223_/Q impaired the ability of Vpu to down-regulate BST-2 from the cell surface, as similar levels of BST-2 were detected at the surface of cells expressing WT and Vpu-defective viruses (Figure 9, upper right panel and Figure 9B). Together with the results obtained from depleting HRS (Figure 8C–D), our data suggest that the integrity of the ESCRT machinery is required for Vpu to down-regulate BST-2 from the cell surface.

### HRS interacts with BST-2 and Vpu

**Figure 10:**
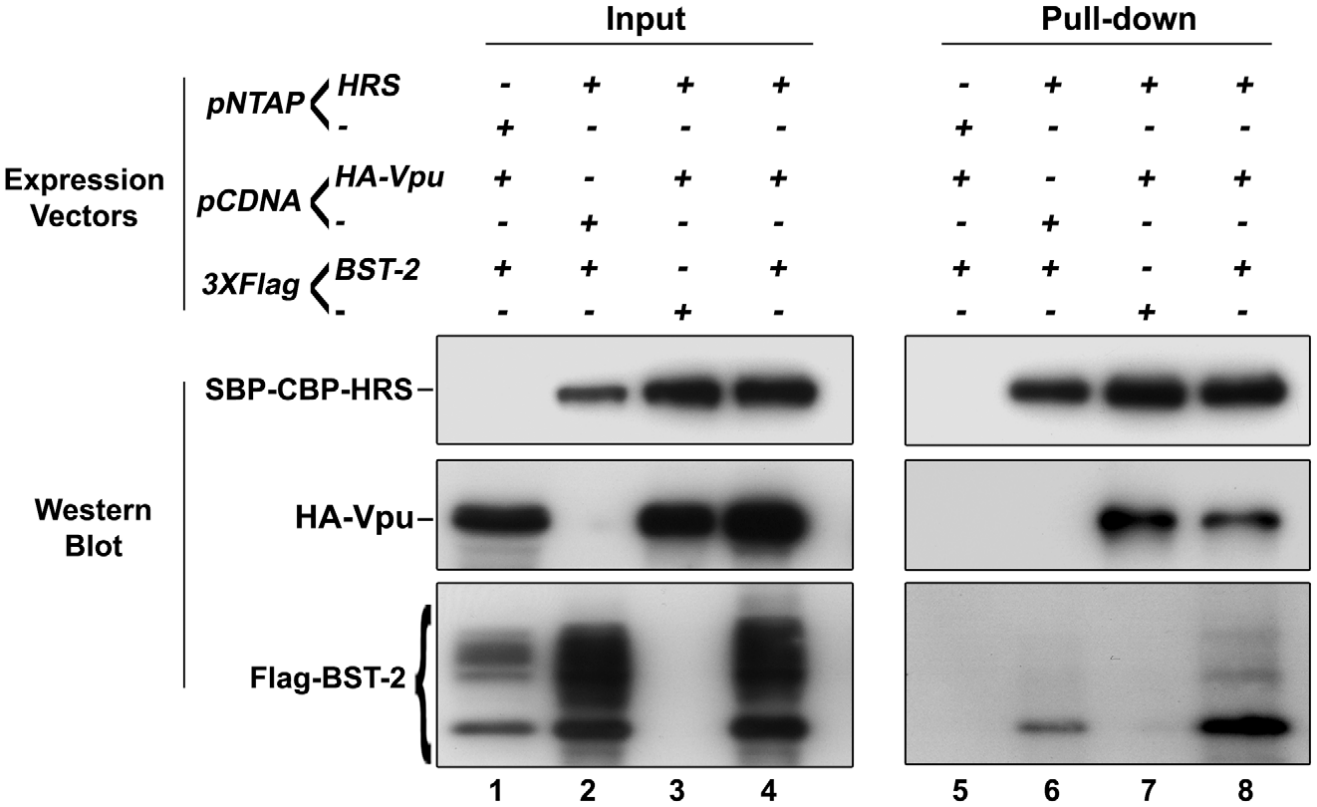
HRS interacts with BST-2 and Vpu. HEK 293T cells were transfected with empty plasmid encoding SBP-CBP (pNTAP-) along with plasmid encoding Flag-tagged BST-2 (Flag-BST-2) and HA-tagged Vpu (HA-Vpu, Lanes 1 and 5) or expression vector for SBP-CBP-tagged HRS (pNTAP-HRS) along with Flag-BST-2 (Lanes 2 and 6); HA-Vpu (Lanes 3 and 7) or both proteins (Lanes 4 and 8). The relative amounts of plasmids transfected were kept constant by adding the corresponding empty vector. Twenty-four hours later the cells were lysed and SBP-CBP-HRS was pulled-down and binding of HA-Vpu and Flag-BST-2 was analysed by western-blot. Left panels represent the input and right panels represent bound proteins. The data is representative of 4 independent experiments.

We showed that HRS is required for the targeting of BST-2 to the degradation pathway (Figure 4) and for the ability of Vpu to down-regulate BST-2 (Figures 5 and 8) to promote efficient release of HIV-1 particles. Since HRS is physiologically involved in the recognition of cargo on endosomal membranes, we postulated that HRS might interact with BST-2 and/or Vpu. To test this, HEK 293T cells were transfected with plasmids encoding HRS fused to SBP-CBP tags (SBP-CBP-HRS) along with Flag-BST-2 and/or HA-Vpu. Interaction with HRS was assessed by pull-down of SBP-CBP-HRS followed by western-blot analyses of bound proteins. Figure 10 shows that HRS interacts with Flag-BST-2 (Figure 10, Lane 6) and is also able to bind HA-Vpu (Figure 10, Lane 7). Interestingly all three proteins co-precipitated when expressed together (Figure 10, Lane 8), and HRS apparently bound more BST-2 in the presence of Vpu. This suggests that Vpu enhances the recognition of BST-2 by the ESCRT machinery, perhaps by bridging BST-2 and HRS.

## Discussion

Studies of the mechanism through which HIV-1 particles are assembled and released from infected cells have revealed a major role for the ESCRT machinery. ESCRT-I and ESCRT-III complexes are recruited to sites of virus assembly by binding to the viral Gag protein, promoting the scission of assembled viral particles [5], [6], [7], [8]. An additional step in the release of mature virus has been highlighted through studies of the HIV-1 accessory protein Vpu. Vpu had been implicated in the efficient release of HIV-1 from certain cell lines (referred to as restrictive cells) but its cellular target was unknown. Recently, BST-2/tetherin was identified as a cellular restriction factor that impedes the release of HIV-1 by physically tethering fully formed mature particles to the plasma membrane of infected cells. It was proposed that, by reducing BST-2/tetherin cell surface expression, Vpu abrogates BST-2/tetherin function, thus allowing efficient virus release [12], [13], [14], [15], [17], [22]. Here we show that the HRS component of the ESCRT-0 complex facilitates Vpu-induced BST-2 down-regulation and degradation, thereby contributing to efficient HIV-1 release. Our data thus highlight an additional role for the ESCRT machinery in HIV-1 release, following ESCRT-mediated membrane scission.

Physiologically, HRS is thought to initiate the ESCRT-mediated formation of MVBs. A PSAP motif in HRS recruits the ESCRT machinery to endosomal membranes through interaction with the ESCRT-I component TSG101 [16]. These events are mimicked by HIV when ESCRT-I is recruited to budding sites through a similar PTAP motif in the C-terminal p6 “late domain” of Gag [6], [9]. Although HRS is not needed to recruit the ESCRT machinery for viral scission [9], we show that it is required to promote efficient Vpu-dependent release of HIV-1 particles following completion of the budding reactions. The reduced HIV-1 release caused by HRS depletion is not a consequence of an indirect effect on the ability of Gag to recruit TSG101, as HRS depletion neither impaired the binding of Gag to TSG101 nor altered Gag processing (Figure 2; [5]). A role for HRS in HIV-1 morphogenesis was previously investigated in HEK 293T cells. In that study, over-expression of HRS or HRS deletion mutants induced a “late budding phenotype” by depleting the pool of free TSG101 available to Gag to promote viral scission [23]. These data are not incompatible with our current results, but use of RNA interference and BST-2 expressing cells (HEK 293T cells do not constitutively express BST-2/tetherin and are non-restrictive for Vpu-defective HIV-1) allowed us to uncover an additional level of ESCRT-mediated regulation of HIV-1 release. A key finding suggesting that HRS might influence virus release was the observation of large clusters of mature virus particles on the surface and in endosomes of HIV-1 infected HRS depleted cells (Figure 3). This phenotype was reminiscent of that observed following infection of BST-2-expressing cells with Vpu-defective HIV-1 [13] and was clearly different from that induced by the absence of TSG101 recruitment to budding sites, which is characterized by the cell surface accumulation of incomplete immature viral buds [5]. This suggested that HRS might contribute to the mechanism counteracting BST-2 restriction of HIV-1 release.

We therefore investigated whether the ESCRT machinery, and in particular HRS and TSG101, facilitate BST-2 down-modulation and degradation. Previous studies have indicated that BST-2 undergoes clathrin-mediated endocytosis from the plasma membrane and traffics through recycling endosomes and the *trans* Golgi network (TGN) [24]. Indeed a significant fraction of BST-2 is located in the TGN at steady state [25], [26]. Here we show that BST-2 undergoes rapid constitutive turnover in HeLa cells (half time < 2 h) that is dependent on both HRS and TSG101, suggesting that although BST-2 cycles between the plasma membrane, endosomes and the TGN, a significant fraction is sorted to lysosomes and degraded. Interference with the ESCRT machinery, and associated effects on lysosomal sorting and degradation, by RNA interference or over-expression of WT or dominant negative proteins of the MVB pathway, resulted in inhibition of constitutive BST-2 degradation and its accumulation in intracellular structures that are likely to be endosomes and/or the TGN (Figure 4 and Figure S5). Thus, the ESCRT machinery, and in particular HRS and TSG101, sorts BST-2 to the ESCRT/MVB degradation pathway and Vpu appears to enhance this degradation, leading to barely detectable levels of BST-2 in HIV-1 infected cells (Figure 8).

Vpu abrogation of the restriction to HIV-1 release is associated with BST-2 down-regulation from the surface of infected cells [11], [12], [15], [17], [22]. To date, the exact mechanism through which Vpu reduces BST-2 expression is not clearly understood. Vpu may decrease BST-2 cell surface expression by increasing its endocytosis and/or by decreasing its recycling to the cell surface, thereby diverting more of the internalized protein to lysosomes. Recent studies reported that Vpu does not increase the rate of BST-2 endocytosis [17] and that BST-2 mutated on two cytosolic domain tyrosine residues (Y6/Y8) involved in its internalization remains sensitive to Vpu [27], [28], [29]. However, inhibition of endocytosis by the dynamin 2-K44A mutant, as well as depletion of the clathrin adaptor AP2, partially impair Vpu-induced BST-2 down-regulation from the plasma membrane [17], [29]. In addition, Vpu accelerates the internalization rate of the BST-2 Y6/Y8 mutant [30], suggesting a role for Vpu in BST-2 endocytosis. Alternatively, other studies suggest that Vpu may slow the transport of BST-2 (both newly synthesized and recycled) to the cell surface and sequester the restriction factor in a perinuclear compartment [27], [31], [32]. This last model is reminiscent of the mechanism by which the Env proteins from HIV-2 and tantalus monkey SIV (SIVtan) overcome BST-2 restriction, whereby BST-2 is redistributed from the cell surface to intracellular compartments, such as recycling endosomes and/or the TGN, without degradation [33], [34], [35]. Finally, it has been proposed that Vpu enhances the release of HIV-1 in the absence of BST-2 cell surface down-regulation and intracellular depletion [21], [36], suggesting another mechanism of Vpu action on BST-2 restriction.

Our data indicate that the integrity of the ESCRT/MVB pathway is required for Vpu to counteract BST-2 restriction. HRS depletion reduces Vpu-induced cell surface down-regulation of BST-2, impairs Vpu-induced BST-2 degradation and reduces HIV-1 release (Figures 1, 8 and S4). Moreover, expression of dominant negative mutant of the AAA-ATPase VPS4 (VPS4-E223Q), another component of the ESCRT machinery, impairs Vpu-induced cell surface down–regulation of BST-2 (Figure 9). Together with observations that inhibitors of the endosomal/lysosomal pathway impair Vpu-induced BST-2 down-regulation [17], [22], our data support a model in which Vpu may sort BST-2 at the level of endosomes, reducing its recycling to the plasma membrane [17], [22]. This model is also in agreement with the redistribution of BST-2 to transferrin receptor positive endosomal compartments upon HIV-1 infection, as described by Habermann et al. [37]. Accordingly, one could postulate that the accumulation of BST-2 in HRS depleted cells exceeds the degradation capacity of Vpu and inhibits the release of wild-type HIV-1 by misrouting BST-2 back to the cell surface.

Vpu downregulates the cell surface expression of BST-2 and increases its degradation [11], [12], [15], [17], [22]. Whether Vpu targets BST-2 for proteasomal or lysosomal degradation is still a matter of debate. Experiments using proteasome inhibitors or a dominant negative mutant of ubiquitin have suggested that Vpu connects BST-2 to the Skp1-cullin-F-Box (SCF) ubiquitin ligase complex and targets it for proteasomal degradation, as described for Vpu-mediated degradation of CD4 [11], [12], [38]. However, prolonged treatment with proteasome inhibitors can deplete cellular pools of free ubiquitin and thereby indirectly affect alternative ubiquitin-dependent sorting and degradation pathways [18]. Studies using inhibitors of the endosomal/lysosomal pathway support a model in which Vpu targets BST-2 for lysosomal degradation [17], [22]. Although our data cannot rule out the involvement of the proteasome pathway, the involvement of HRS and the ESCRT machinery in both constitutive and Vpu-enhanced degradation of BST-2 strongly supports a role for lysosomal degradation in BST-2 down regulation. How Vpu targets BST-2 to the MVB pathway is also unclear. Studies have reported that Vpu interacts with BST-2 through the transmembrane domains of both proteins [27], [29] and endosomal co-localisation of BST-2 with Vpu has been described [13], [15]. Interestingly, we showed that HRS interacts with BST-2 and Vpu (Figure 10). One model to explain these observations is that through binding with BST-2 [27], [29] and HRS, Vpu might stabilize BST-2 on endosomal membranes and, by recruiting the β-TRCP2-Skp1-cullin E3 ubiquitin ligase complex, allow the efficient ubiquitination of BST-2 to permit its recognition by the ESCRT machinery through HRS, as discussed in a recent study [17]. Indeed, recent studies have shown that β-TRCP2, a subunit of the Skp1-cullin1-F-Box (SCF) ubiquitin ligase complex is required for Vpu to down-regulate BST-2 expression [12], [17], [22], [29] and the SCF ubiquitin ligase complex can be recruited to endocytic membranes [39]. Moreover, it has been described that certain receptors, such as the growth hormone receptor (GHR) and the interferon α receptor (IFNAR1), are targeted for lysosomal degradation following their binding to β-TRCP2 and subsequent ubiquitination [40], [41].

Physiologically, HRS is involved in the recognition of ubiquitinylated protein flagged for lysosomal degradation. BST-2 was recently shown to be ubiquitinylated and Vpu expression was shown to increase this ubiquitination [42], [43]. BST-2 contains two lysine residues, the common target of ubiquitination, in its cytosolic N-terminal domain at positions 18 and 21. Mutation of these residues does not impair BST-2 sensitivity to Vpu-mediated down-regulation, suggesting that BST-2 ubiquitination is not required for Vpu to target BST-2 to the MVB degradation pathway [12], [28], [42]. However, residues other than lysine may undergo ubiquitination (e.g. cysteine, threonine and serine residues). Ubiquitination of lysine and serine/threonine residues in the cytosolic domain of CD4 has been shown to be required for its targeting to the degradation pathway by Vpu [44]. BST-2 contains a threonine and two serine residues at positions 3 to 5 and, interestingly, ubiquitination of BST-2 on these residues was recently shown to be involved in Vpu-induced release of viral particles [43]. These observations together with indirect evidence from the use of drugs that affect cellular ubiquitin pools, suggest a role for ubiquitin in Vpu-induced targeting of BST-2 to the degradation pathway [11], [12], [17], [43]. Further analyses will be necessary to decipher the exact role of HRS in targeting BST-2 to the MVB pathway under physiological conditions and after infection by HIV-1, and the requirement for ubiquitination in these processes. Moreover, it would be interesting to further establish the role of this pathway in Vpu's function in the natural cellular targets of HIV-1, such as primary CD4 T-cells and macrophages.

In summary, we show that HRS facilitates the constitutive turnover of BST-2/tetherin and that Vpu enhances this degradation. HRS is required for Vpu to efficiently down-regulate BST-2 from the surface of the cells and to target BST-2 to the MVB/lysosomal degradation pathway, thereby promoting HIV-1 release. This highlights a role for HRS and the ESCRT/MVB pathway in the regulation of Vpu function. Although the precise molecular and cellular mechanisms by which Vpu connects to the ESCRT machinery to modulate BST-2 expression remains to be elucidated, our data bring significant new insights to understanding how this viral protein uses cellular machineries to counteract host cell restriction and favour HIV-1 dissemination.

## Materials and Methods

### Recombinant DNA procedures

HRS and VPS4 cDNA were cloned into pEGFP-C vector (Clontech, France). The VPS4 E223Q mutant was made by PCR mutagenesis using the QuikChange II site directed mutagenesis kit (Stratagene, France). Expression vectors for HRS fused to the Streptavidin binding protein (SBP) and Calmodulin binding protein (CBP) affinity tags were obtained by cloning HRS into the pNTAP-B vector (Stratagene, France). The expression vector for Flag-tagged BST-2 was obtained by cloning of BST-2 into p3XFlag vector. cDNA for HA tagged-Vpu cloned into pCDNA3.1 was a gift from Dr. Florence Margottin-Goguet [45] All mutagenesis and subclonings were verified by DNA sequencing.

### Cell culture and transfection

HeLa, HeLa P4R5 MAGI (NIH; NIH AIDS Research and Reference Reagent Program, Division of AIDS, NIAID), HeLa TZM-bl (NIH) and HEK 293T cells were grown in DMEM plus glutamine, antibiotics and 10% decomplemented-FCS (foetal calf serum) (GibcoBRL, Invitrogen). HeLa P4R5 MAGI cell cultures were supplemented with 100 µg/mL geneticin and 1 µg/mL puromycin.

The 21-nucleotide RNA duplexes designed to target HRS (5′ CGACAAGAACCCACACGUCdTdT 3′ at positions 162-180) or TSG101 (5′ CCUCCAGUCUUCUCUCGUCdTdT 3′ at positions 415-433) were previously described [5], [16] and synthesized by Thermo Scientific Dharmacon (Perbio Science, France). The On-target *plus* SMART-pool siRNA targeting BST-2 was purchased from Dharmacon (# L-011817-00). The On-target *plus* non-targeting siRNA 1 (# D-001810-01 from Dharmacon) was used as control. The cells were transfected with 4 to 30 nM siRNA using Lipofectamine RNAiMAX (Invitrogen) according to the manufacturer's instructions.

Transient transfections of HeLa and HEK 293T cells with mammalian expression vectors were performed using FuGENE (Roche diagnostics) following the manufacturer's instructions.

### Cycloheximide treatment

Cells were pre-incubated for 45 min with DMEM plus 10 mM Hepes, 1 mg/ml BSA (Calbiochem, VWR) and 10 µg/ml Cycloheximide (Calbiochem). The cells were then washed and incubated for the indicated times in regular growth medium plus 10 mM Hepes, 10 µg/ml cycloheximide and 150 ng/ml Epidermal Growth Factor (EGF) (Invitrogen) where indicated. The cells were harvested, washed in PBS, lysed and analysed by western blotting as described [46].

### Viral stocks

Stocks of HIV-1 NL4-3 WT (NIH), HIV-1 NL4-3 Udel [47], VSV-G pseudotyped NL4-3 WT and VSV-G pseudotyped NL4-3 Udel were obtained as described [46]. Viral titres (Multiplicity of Infection: MOI) were assessed by infection of the indicator cells HeLa TZM-bl (bearing the β-galactosidase gene under the control of HIV-1 LTR) with serial dilutions of the stocks, followed by a β-galactosidase coloration of the cells and counting of blue cells.

### HIV-1 replication assay

HeLa P4R5 cells (1×10^5^) transfected with siRNA were infected with NL4-3 HIV-1 at an MOI of 0.005. Six hours after infection, cells were washed and placed in fresh medium. Supernatants were collected every 24 h for 4 days and used for HIV-1 CAp24 quantification by ELISA. At the end of the experiment, cells were lysed and HRS depletion was assessed by western blotting using rabbit anti-HRS (Euromedex, France) and mouse anti-tubulin (DM1A, Sigma, France) antibodies.

### HIV-1 production, infectivity and incorporation assays

For HIV-1 production assays on a single round of replication, HeLa cells or 293T cells were treated with siRNA as described above. After 48 h, cells were infected with NL4-3 (WT or Udel) HIV-1 pseudotyped with VSV-G for 6 hours at an MOI of 0.5. The cells were washed 24 h later, and incubated for a further 24 h. Supernatants were then collected, 0.45 µm-filtered, and used for HIV-1 CAp24 quantification by ELISA (released CAp24). Viral particles released into the supernatant were pelleted through a 20% sucrose cushion by ultracentrifugation at 150 000 g for 90 min, and resuspended in Laemmli buffer. Pelleted viruses were analysed by western blotting using mouse anti-CAp24 (ARP366, NIBSC). The cell lysates were analyzed by western blotting using mouse anti-CAp24 (ARP366, National Institute for Biological Standards and Control (NIBSC), UK), mouse anti-MAp17 (18A, Hybridolab, France), mouse anti-SUgp120 (110H, Hybridolab), human anti-TMgp41 (2F5, NIH), rabbit anti-Vpu (NIH), rabbit anti-Nef (NIH), rabbit anti-HRS (Euromedex France, Souffelweyersheim, France), mouse anti-TSG101 (BD-Biosciences, France), rabbit anti-BST-2 (NIH) and mouse anti-tubulin antibodies. HIV-1 CAp24 antigen contained in the cell lysates (cell-associated CAp24) was also quantified by ELISA.

In a single round infectivity assay, the titres of released viruses were determined by infection of the indicator cells HeLa TZM-bl in a standardized 96-well titration assay by luminometric analysis of firefly luciferase activity (Kit luciferase Assay reagent, Promega, France) following the manufacturer's instructions.

### GST-pull down experiments

The GST-pull down experiment was done using cytosolic extracts of siRNA transfected HeLa cells prepared in 50 mM Tris pH 7.6, 150 mM NaCl, 2 mM EDTA, 0.5% (v/v) Triton X-100 (lysis buffer). Aliquots (2 µg) of purified GST, GST-p6 or GST-Gag proteins were immobilized on glutathione-Sepharose beads (GE healthcare) and incubated for 4 h with 2 mg of cell extract (diluted in 500 µl of lysis buffer). The beads were then washed 3 times in lysis buffer and once in PBS, and bound cellular proteins were separated by SDS-PAGE and revealed by western blotting using antibodies to TSG101, HRS and tubulin.

### Tap-Tag experiments

HEK 293T cells were transfected with (a SBP-CBP-tagged HRS expression vector (pNTAP-HRS) or the corresponding empty vector (pNTAP) along with HA-tagged Vpu and Flag-tagged BST-2 expression vectors or with (b) SBP-CBP-tagged HRS expression vector along with HA-tagged Vpu and p3X-Flag empty vector or (c) Flag-tagged BST-2 expression vector and PCDNA3-1 empty vector. Cells were harvested 24 hours after transfection and lysed following instructions provided by the Interplay mammalian TAP-system (Stratagene, France). The cell lysates were cleared by centrifugation and subjected to two consecutive rounds of pull-down by incubation with a Streptavidin resin, followed by the elution of the bound cellular proteins and incubation of the eluted protein with a Calmodulin resin (following the manufacturer's instructions). The beads were then washed in lysis buffer and the TAP-purified proteins were resolved by SDS-PAGE and revealed by western blotting using antibodies to HRS, HA and Flag.

### Flow cytometry

Forty eight hours post-infection, siRNA transfected HeLa cells were harvested by scrapping, washed twice in cold PBS/1% (w/v) BSA and stained for 1 hour at 4°C with rabbit anti-BST-2 antibody (NIH) or control rabbit IgGs (rabbit serum; Sigma, France) in PBS/1%BSA. The cells were then washed three times in PBS/1%BSA, and stained for 1 hour at 4°C with a cy5-conjugated donkey anti-rabbit antibody in PBS/1%BSA. Cells were washed, fixed in 4% paraformaldehyde (PFA), quenched for 10 min in PBS/0.1 M glycine, and then permeabilized in PBS/1% BSA/0.05% saponin before staining with a FITC-conjugated anti-CAp24 (KC57-FITC, Beckman Coulter, France). Cells were washed and fixed in PBS/1% BSA/1% PFA before analysis using the Cytomics FC500 Flow Cytometer (Beckman Coulter). Gates for FITC were set using non-infected cells.

HeLa cells transfected with plasmids encoding the GFP-tagged constructs along with plasmid encoding the provirus NL4-3 wt or Vpu-defective NL4-3 (NL4-3 Udel) were harvested 48 hours after transfection and stained for 1 hour at 4°C with anti-BST-2 mouse monoclonal antibody (Abnova, Tebu-bio; France) or isotype control mouse IgG1 (BD-bioscience; France), along with human anti-Env antibody (2G12, NIBSC), in PBS/1%BSA. The cells were then washed three times in PBS/1% BSA, and stained for 1 hour at 4°C with an Alexa 647-conjugated donkey anti-mouse and PE-conjugated donkey anti-human antibodies in PBS/1% BSA. Cells were washed, fixed in PBS/1% BSA/1% PFA before analysis using the Cytomics FC500 Flow Cytometer. Gates for GFP and PE were set using respectively non-transfected and non-infected cells. All the data were analysed using the CXP cytometer software.

### Immunofluorescence microscopy

Cells, grown on cover slips, were permeabilized in PBS/0.1% BSA/0.05% saponin [16] before fixation with 4% PFA in PBS for 15 min. PFA-fixed cells were permeabilized and blocked with 0.2% BSA/0.1% saponin in PBS for 30 min. Cells were incubated for 30 min with mouse anti-BST-2 antibody (Abnova, France) alone or together with human anti-Env antibody (2G12, NIH) in blocking solution, washed and incubated for 30 min with appropriate fluorophore-conjugated secondary antibodies. Cells were analyzed with a Leica SP2 confocal microscope. Series of optical sections were recorded and image processing was performed using Adobe Photoshop CS2 software.

### Labeling of cryosections for immunofluorescence or EM

Cells were fixed in 4% PFA in 0.1 M sodium phosphate buffer, pH 7.4, embedded in gelatine, and frozen for cryosectioning, as described previously [48], [49]. For immunofluorescence staining, semi-thin (0.5 µm) cryosections were placed on glass slides, quenched in 50 mM glycine/50 mM NH_4_Cl and extracted for 6 min in 0.1% Triton X-100. Sections were stained with antibodies to MAp17 (4C9, ARP 342, NIBSC) and Env (2G12, NIBSC) and goat anti-mouse Alexafluor-488 and goat anti-human-Alexafluor-594 (Invitrogen) diluted in PBS/1% BSA, and mounted in Mowiol. Sections were examined with an Axioskop (Carl Zeiss MicroImaging, Inc.); images were recorded with a CCD camera (Orca C4742-95; Hamamatsu) and processed using Adobe Photoshop 8.

For immuno-EM, ultrathin cryosections (50 nm) were stained with mouse antibodies against HIV-1 p24/p55 (ARP365 and ARP366, NIBSC), a rabbit anti-mouse bridging antibody (DakoCytomation, Ely, UK), and 5 nm PAG (Protein A gold reagents were obtained from the EM Lab, Utrecht University, Utrecht, The Netherlands). Sections were fixed in 1% (v/v) glutaraldehyde for 10 min, quenched in 50 mM glycine-50 mM NH4Cl in PBS and incubated with the human anti-Env mAb 2G12 and 10 nm PAG. Sections were embedded in uranyl acetate in methylcellulose, as described previously [49], and examined with a Technai G2 Spirit transmission electron microscope (FEI Company UK. Ltd., Cambridge, UK). Digital images were recorded with a Morada 11 MegaPixel TEM camera (Soft Imaging System) and the AnalySIS software package. Images were adjusted for brightness and contrast, and figures were assembled with Photoshop 8.

## Supporting Information

Figure S1 HRS depletion does not affect entry of HIV-1 in HeLa P4R5 cells. HeLa P4R5 cells, transfected with either control siRNA (CT) or siRNA targeting HRS, were infected with NL4-3 HIV-1 WT at a low MOI = 0.001. Cells were washed 4 h later and placed in fresh medium supplemented with AMD3100, a CXCR4 inhibitor. After 24 h, the cells were fixed with 0.5% glutaraldehyde in PBS and infected cells revealed by X-gal coloration (potassium ferrocyanide hydrate 4 mM, potassium ferricyanide 4 mM, MgCl_2_ 2 mM, X-Gal (5-bromo-4-chloro-3-indolyl β-D-galactopyranoside) 0.4 mg/ml). Blue coloured cells were counted in duplicate and values were normalized to those obtained for the control cells (set as 100%). Bars represent the mean −/+ SD from 3 independent experiments. CT/NI corresponds to non-transfected, non-infected cells.
(0.17 MB TIF)

Figure S2 Binding of TSG101 to Gag. Lysates of HeLa cells transfected with either control siRNA (CT) or siRNA targeting HRS or TSG101 were incubated with equal amounts of purified GST (lanes 4 to 6) or GST-Gag (lanes 8 to 10). TSG101 binding and HRS depletion were analysed by western blotting (upper panels). Tubulin is the loading control for cellular proteins. Lower panel: Ponceau red staining of the membrane used for western blotting. These data are representative of 3 independent experiments.
(0.46 MB TIF)

Figure S3 HRS depletion decreases Env incorporation in nascent viral particles. HeLa cells transfected with either control siRNA (CT) or siRNA targeting HRS, were infected with VSV-G pseudotyped wt NL4-3 HIV-1 (NL4-3 WT). Supernatants of infected cells were harvested 48 h later, 0.45 µm-filtered, and the virions pelleted through a 20% sucrose cushion by ultracentrifugation at 150 000 g for 90 min. The virus pellets were resuspended in Laemmli buffer. Cell lysates and pelleted viruses were analysed by western blotting using mouse anti-CAp24 (ARP366, NIBSC), mouse anti-TMgp41 (41A, Hybridolab) and anti-tubulin antibodies. Left panels represent loading of equal amounts of cell proteins (to visualize the intracellular accumulation of viral proteins in HRS depleted cells). Middle panels represent loading of equal volumes of the virus samples (to visualize the decrease of virus release in HRS depleted cells). Left panels represent loading of equal amounts of viral particles (equal amount of CAp24; to visualize Env content for a fixed amount of viral particles).
(0.34 MB TIF)

Figure S4 Further immuno-EM images of control HeLa cells or cells treated with siRNA for Hrs. HeLa cells were transfected with either siRNA control (A, B) or siRNA targeting HRS (C - F). After 48 h, the cells were infected with NL4-3 HIV-1 pseudotyped with VSV-G and, after a further 48 h, fixed and prepared for cryosectioning. Ultrathin (50 nm) cryosections were double labelled with antibodies against Gag p24/p55 (5 nm PAG) and anti-Env 2G12 (10 nm PAG). Infected cells were identified by the scattered 5 nm gold particles (Gag p55) over the cytoplasm (e.g. as indicated by the arrows). The Env protein (10 nm PAG, e.g. at the arrowheads) was seen over membranes near the Golgi apparatus (referred as G, see panels A and C). In control siRNA-treated cells, some mature virus particles labelled with both 5 nm and 10 nm PAG particles are seen at the cell surface (B). In cells treated with HRS siRNA, the extracellular virus clusters were more prominent (D, E), and virus particles were also seen in intracellular vacuoles resembling endosomes (En, see panel F). Mv identifies microvillar protrusions, while PM marks the plasma membrane. Scale bars  = 200 nm.
(9.91 MB TIF)

Figure S5 Analysis of BST-2 expression in HRS and TSG101 depleted cells. (A) Western blot analysis of BST-2 expression in HRS depleted cells: HeLa cells were transfected with control siRNA (CT) or siRNA targeting HRS. Four days after transfection, cells were lysed and equivalent amounts of each sample were analysed by western blotting using antibodies against BST-2, HRS and tubulin as a loading control. (B) Western blot analysis of BST-2 expression in TSG101 depleted cells: HeLa cells were transfected with either control siRNA (CT) or siRNA targeting TSG101. Three days after transfection, cells were lysed and equivalent amounts of each sample were analysed by western blotting using antibodies against BST-2, TSG101 and tubulin as a loading control. (C) Immunofluorescence analysis of BST-2 expression in HRS and TSG101 depleted cells: HeLa cells were transfected with either control siRNA (CT) or siRNA targeting HRS or TSG101. Cells were permeabilized before fixation and then stained with mouse polyclonal antibody against BST-2 and rabbit polyclonal antibody against HRS, followed by staining with Alexa488 conjugated anti-mouse IgG or Alexa594 conjugated anti-rabbit IgG, respectively. Cells were imaged by confocal laser scanning microscopy. Bar: 10 µm.
(1.15 MB TIF)

Table S1 HRS depletion impairs Vpu-induced cell surface down regulation of BST-2. Hela cells transfected with control siRNA (CT) or a siRNA targeting HRS were infected with VSV-G pseudotyped NL4-3 HIV-1. Forty eight hours later the cells were stained for cell surface BST2 followed by an intracellular staining of the Gag product CAp24. Data represent the level of cell surface expression of BST2 (expressed in MFI) in CAp24 negative cells (gate B1) vs. CAp24 positive cells (gate B2) from 3 independent experiments. These data were used to draw the bar graph in Figure 8D.
(0.20 MB TIF)
