# Supplementary figures and images for: The ESCRT-0 Component HRS is Required for HIV-1 Vpu-Mediated BST-2/Tetherin Down-Regulation
Source: PLoS Pathog. 2011 Feb 3;7(2):e1001265. doi: 10.1371/journal.ppat.1001265 (PMC3033365; doi:10.1371/journal.ppat.1001265)

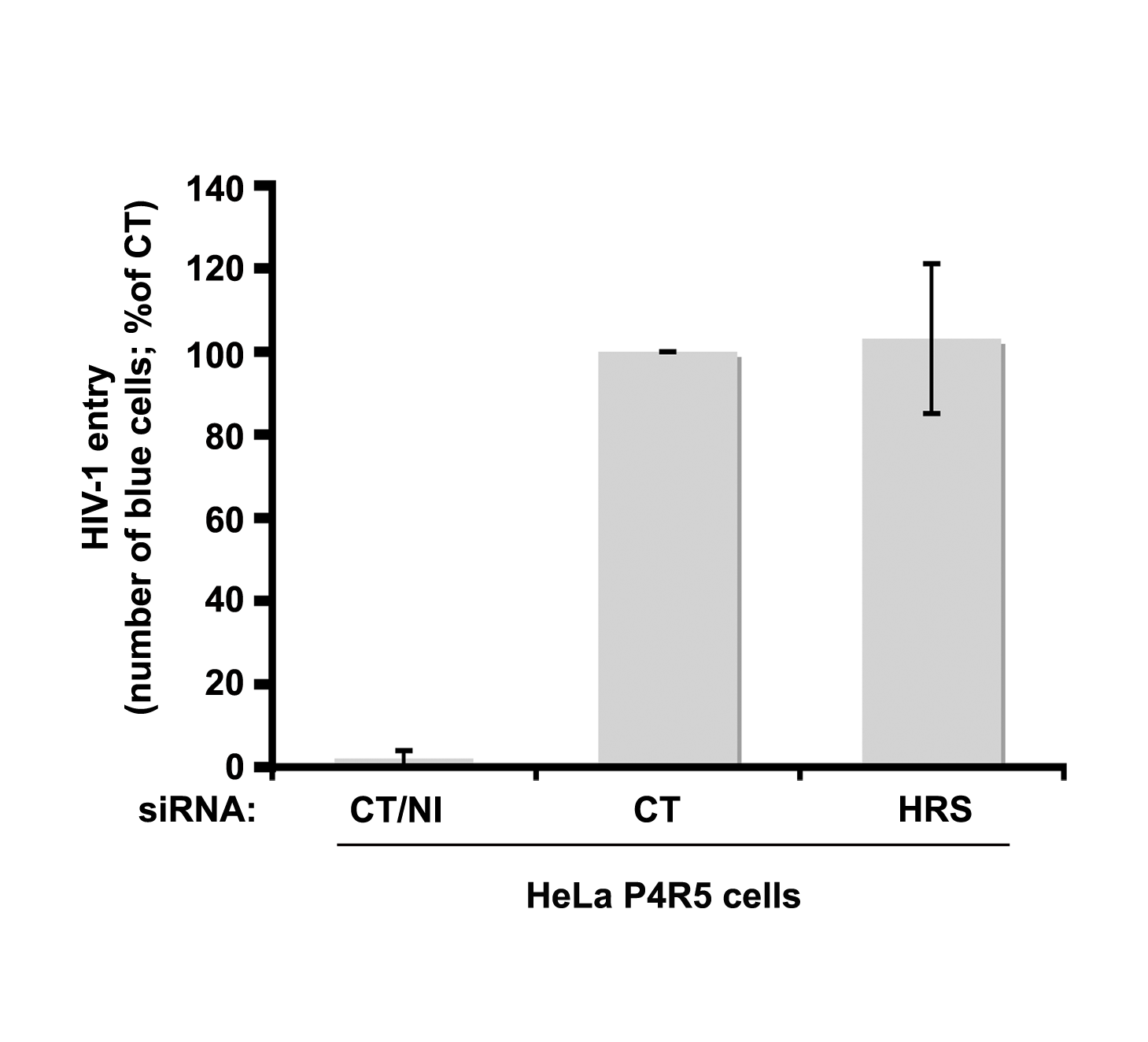

Supplement: Figure S1 — HRS depletion does not affect entry of HIV-1 in HeLa P4R5 cells. HeLa P4R5 cells, transfected with either control siRNA (CT) or siRNA targeting HRS, were infected with NL4-3 HIV-1 WT at a low MOI = 0.001. Cells were washed 4 h later and placed in fresh medium supplemented with AMD3100, a CXCR4 inhibitor. After 24 h, the cells were fixed with 0.5% glutaraldehyde in PBS and infected cells revealed by X-gal coloration (potassium ferrocyanide hydrate 4 mM, potassium ferricyanide 4 mM, MgCl2 2 mM, X-Gal (5-bromo-4-chloro-3-indolyl β-D-galactopyranoside) 0.4 mg/ml). Blue coloured cells were counted in duplicate and values were normalized to those obtained for the control cells (set as 100%). Bars represent the mean −/+ SD from 3 independent experiments. CT/NI corresponds to non-transfected, non-infected cells. (0.17 MB TIF) [file ppat.1001265.s001.tif]

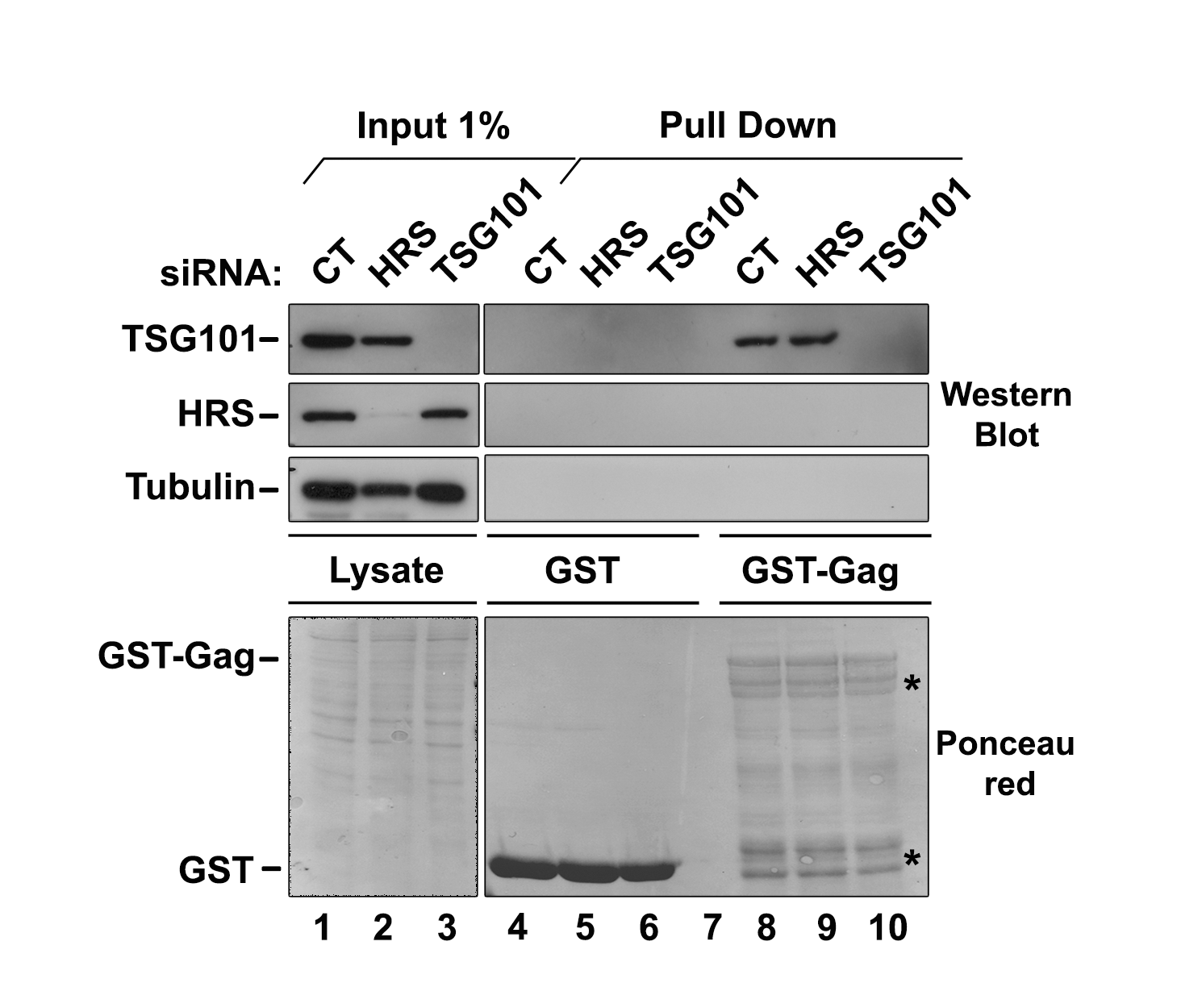

Supplement: Figure S2 — Binding of TSG101 to Gag. Lysates of HeLa cells transfected with either control siRNA (CT) or siRNA targeting HRS or TSG101 were incubated with equal amounts of purified GST (lanes 4 to 6) or GST-Gag (lanes 8 to 10). TSG101 binding and HRS depletion were analysed by western blotting (upper panels). Tubulin is the loading control for cellular proteins. Lower panel: Ponceau red staining of the membrane used for western blotting. These data are representative of 3 independent experiments. (0.46 MB TIF) [file ppat.1001265.s002.tif]

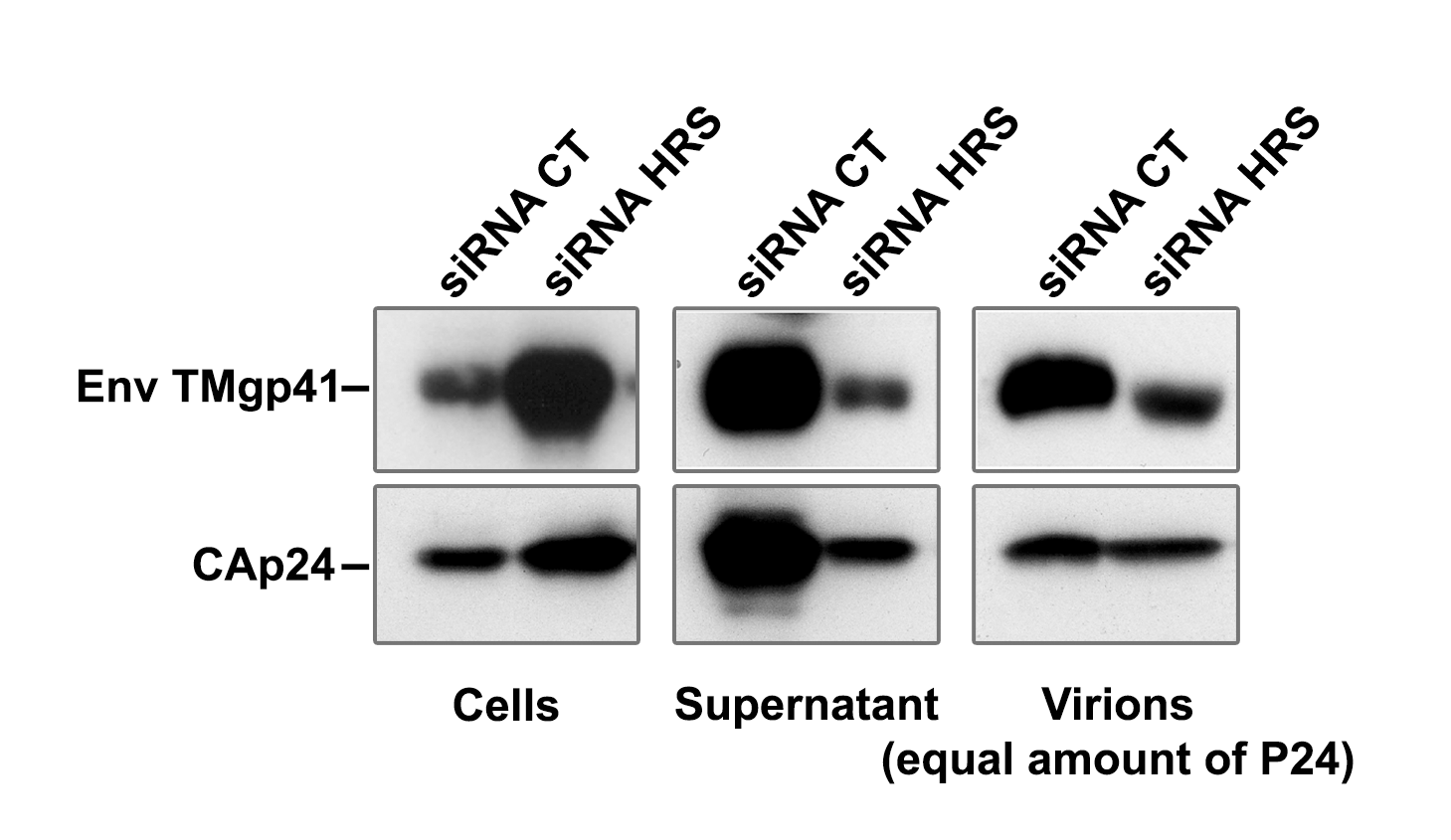

Supplement: Figure S3 — HRS depletion decreases Env incorporation in nascent viral particles. HeLa cells transfected with either control siRNA (CT) or siRNA targeting HRS, were infected with VSV-G pseudotyped wt NL4-3 HIV-1 (NL4-3 WT). Supernatants of infected cells were harvested 48 h later, 0.45 µm-filtered, and the virions pelleted through a 20% sucrose cushion by ultracentrifugation at 150 000 g for 90 min. The virus pellets were resuspended in Laemmli buffer. Cell lysates and pelleted viruses were analysed by western blotting using mouse anti-CAp24 (ARP366, NIBSC), mouse anti-TMgp41 (41A, Hybridolab) and anti-tubulin antibodies. Left panels represent loading of equal amounts of cell proteins (to visualize the intracellular accumulation of viral proteins in HRS depleted cells). Middle panels represent loading of equal volumes of the virus samples (to visualize the decrease of virus release in HRS depleted cells). Left panels represent loading of equal amounts of viral particles (equal amount of CAp24; to visualize Env content for a fixed amount of viral particles). (0.34 MB TIF) [file ppat.1001265.s003.tif]

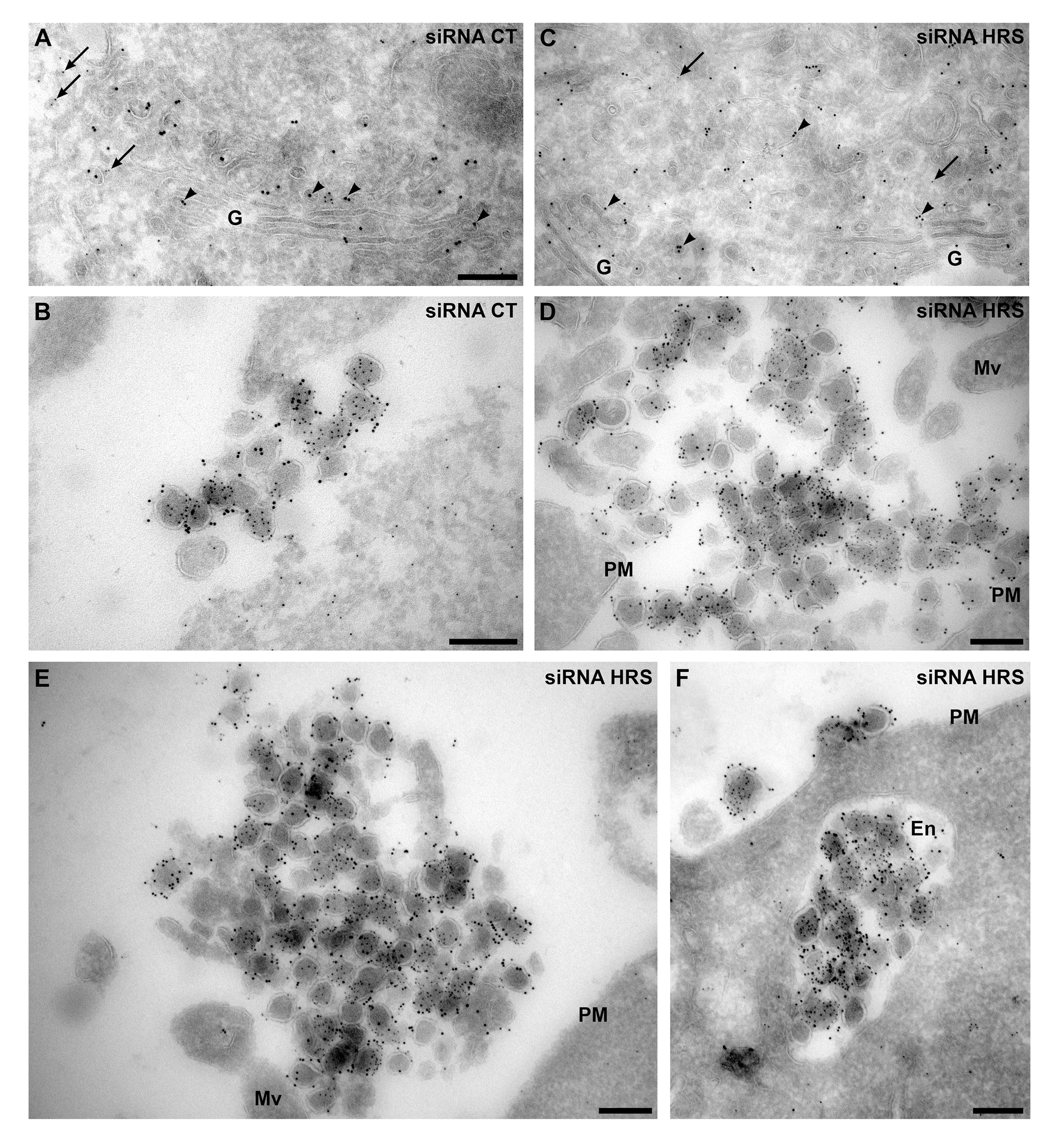

Supplement: Figure S4 — Further immuno-EM images of control HeLa cells or cells treated with siRNA for Hrs. HeLa cells were transfected with either siRNA control (A, B) or siRNA targeting HRS (C - F). After 48 h, the cells were infected with NL4-3 HIV-1 pseudotyped with VSV-G and, after a further 48 h, fixed and prepared for cryosectioning. Ultrathin (50 nm) cryosections were double labelled with antibodies against Gag p24/p55 (5 nm PAG) and anti-Env 2G12 (10 nm PAG). Infected cells were identified by the scattered 5 nm gold particles (Gag p55) over the cytoplasm (e.g. as indicated by the arrows). The Env protein (10 nm PAG, e.g. at the arrowheads) was seen over membranes near the Golgi apparatus (referred as G, see panels A and C). In control siRNA-treated cells, some mature virus particles labelled with both 5 nm and 10 nm PAG particles are seen at the cell surface (B). In cells treated with HRS siRNA, the extracellular virus clusters were more prominent (D, E), and virus particles were also seen in intracellular vacuoles resembling endosomes (En, see panel F). Mv identifies microvillar protrusions, while PM marks the plasma membrane. Scale bars = 200 nm. (9.91 MB TIF) [file ppat.1001265.s004.tif]

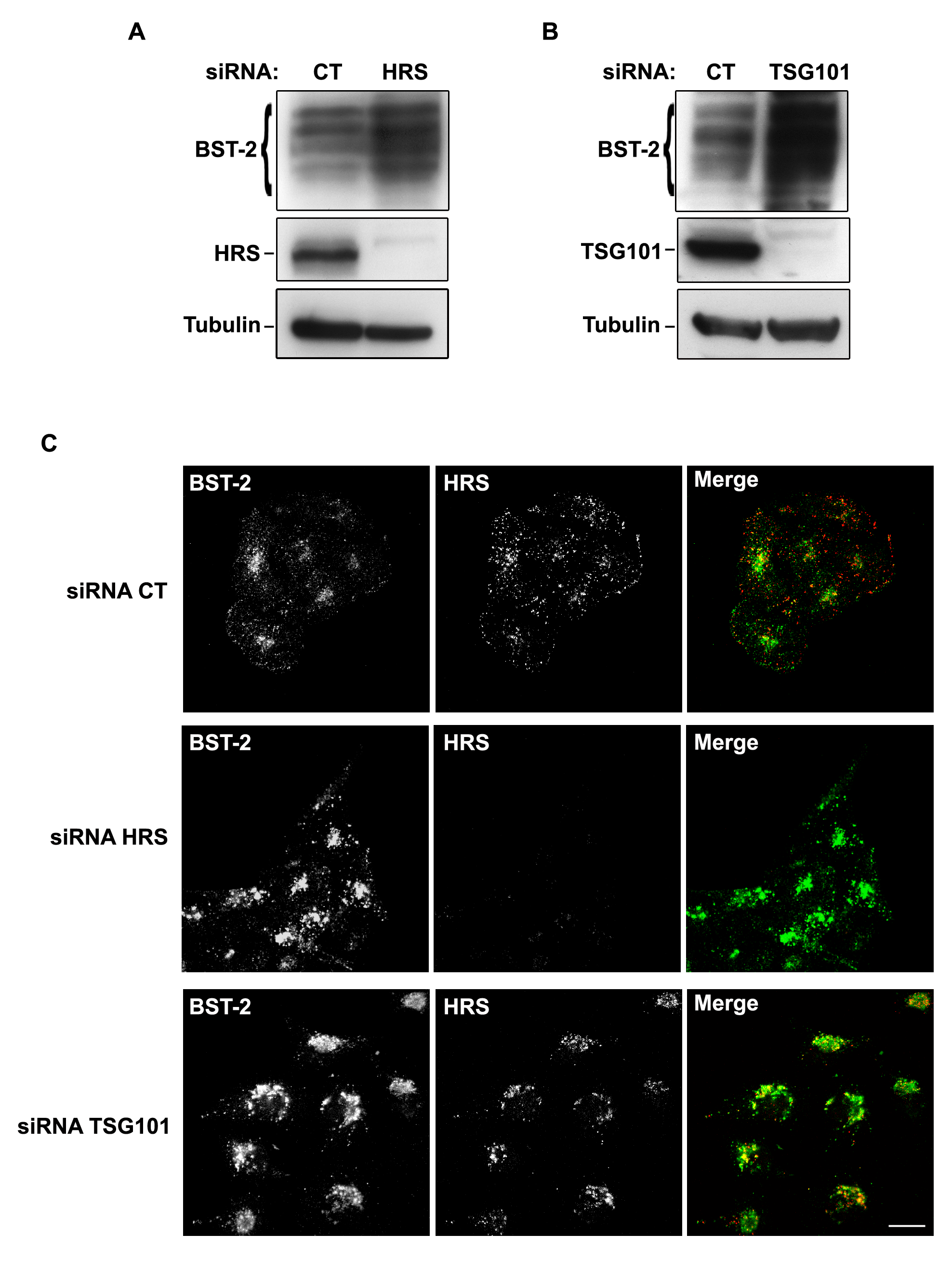

Supplement: Figure S5 — Analysis of BST-2 expression in HRS and TSG101 depleted cells. (A) Western blot analysis of BST-2 expression in HRS depleted cells: HeLa cells were transfected with control siRNA (CT) or siRNA targeting HRS. Four days after transfection, cells were lysed and equivalent amounts of each sample were analysed by western blotting using antibodies against BST-2, HRS and tubulin as a loading control. (B) Western blot analysis of BST-2 expression in TSG101 depleted cells: HeLa cells were transfected with either control siRNA (CT) or siRNA targeting TSG101. Three days after transfection, cells were lysed and equivalent amounts of each sample were analysed by western blotting using antibodies against BST-2, TSG101 and tubulin as a loading control. (C) Immunofluorescence analysis of BST-2 expression in HRS and TSG101 depleted cells: HeLa cells were transfected with either control siRNA (CT) or siRNA targeting HRS or TSG101. Cells were permeabilized before fixation and then stained with mouse polyclonal antibody against BST-2 and rabbit polyclonal antibody against HRS, followed by staining with Alexa488 conjugated anti-mouse IgG or Alexa594 conjugated anti-rabbit IgG, respectively. Cells were imaged by confocal laser scanning microscopy. Bar: 10 µm. (1.15 MB TIF) [file ppat.1001265.s005.tif]

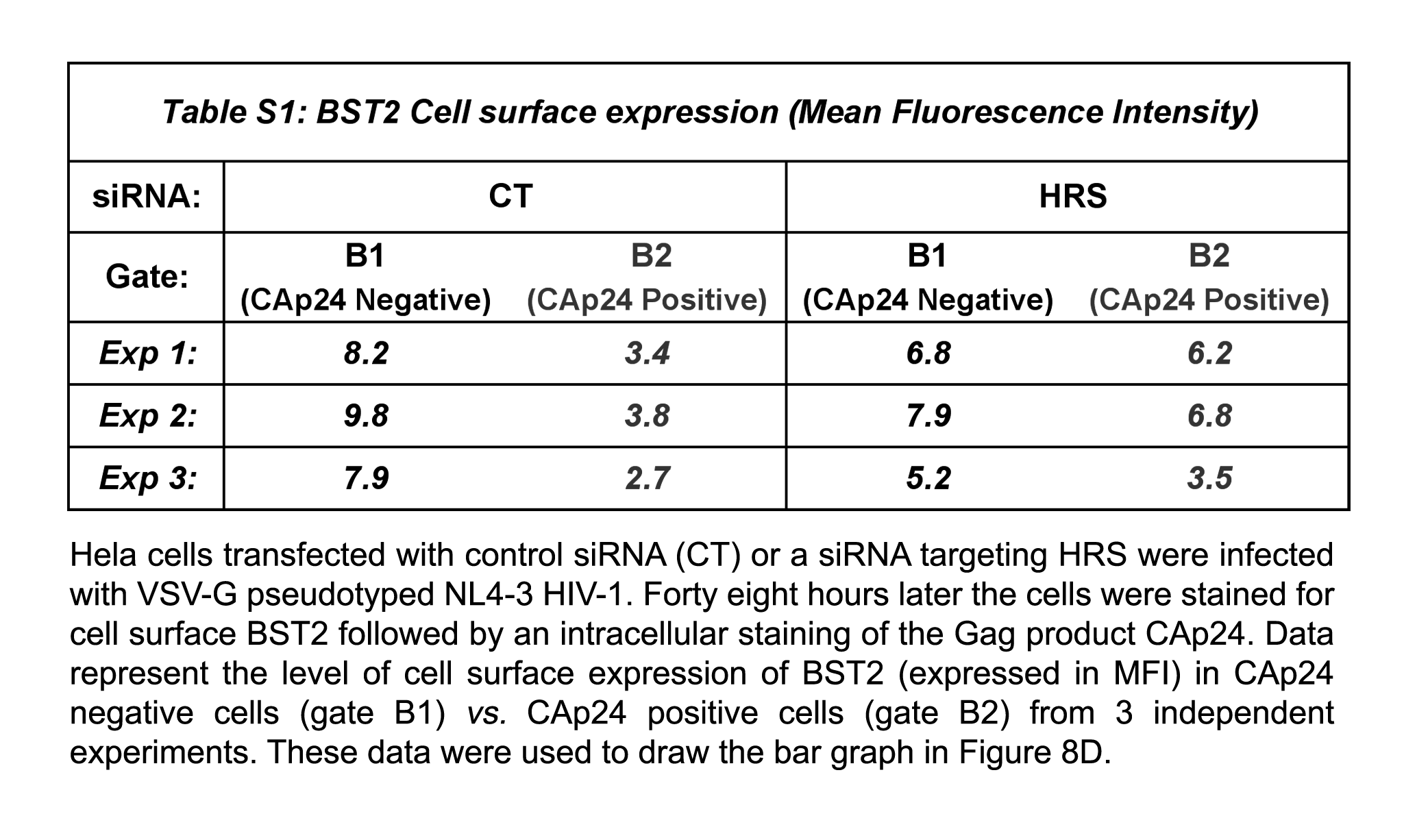

Supplement: Table S1 — HRS depletion impairs Vpu-induced cell surface down regulation of BST-2. Hela cells transfected with control siRNA (CT) or a siRNA targeting HRS were infected with VSV-G pseudotyped NL4-3 HIV-1. Forty eight hours later the cells were stained for cell surface BST2 followed by an intracellular staining of the Gag product CAp24. Data represent the level of cell surface expression of BST2 (expressed in MFI) in CAp24 negative cells (gate B1) vs. CAp24 positive cells (gate B2) from 3 independent experiments. These data were used to draw the bar graph in Figure 8D. (0.20 MB TIF) [file ppat.1001265.s006.tif]
